# Hispano-Americans in Europe: what do we know about their health status and determinants? A scoping review

**DOI:** 10.1186/s12889-015-1799-x

**Published:** 2015-05-07

**Authors:** Maria Roura, Andreu Domingo, Juan M Leyva-Moral, Robert Pool

**Affiliations:** 1 grid.5841.80000000419370247ISGlobal, Barcelona Ctr. Int. Health Res. (CRESIB) Hospital Clínic, Universitat de Barcelona, Rosselló 132, 4th floor, 08036 Barcelona, Spain; 2 grid.7080.fCentre for Demographic Studies, Autonomous University of Barcelona, Carrer de Ca n’Altayó. Edifici E2, Bellaterra, Barcelona, 08193 Spain; 3 grid.5612.00000000121722676Escola Superior d’Infemeria del Mar, University Pompeu Fabra, Doctor Aiguader, 80, Barcelona, Spain; 4 grid.7177.60000000084992262Social Science and Global Health, Centre for Social Science and Global Health, University of Amsterdam, Postbus 15718, 1001 NE Amsterdam, The Netherlands

**Keywords:** Migrants’ health, Ethnic minorities, Population movements, Mobility and health, Social sciences, Social epidemiology, Hispano-Americans, Latin-Americans, Latinos’ health, Research agenda, Social determinants of health, Europe

## Abstract

**Background:**

Policy makers and health practitioners are in need of guidance to respond to the growing geographic mobility of Hispano-American migrants in Europe. Drawing from contributions from epidemiology, social sciences, demography, psychology, psychiatry and economy, this scoping review provides an up-to-date and comprehensive synthesis of studies addressing the health status and determinants of this population. We describe major research gaps and suggest specific avenues of further inquiry.

**Methods:**

We identified systematically papers that addressed the concepts “health” and “Hispano Americans” indexed in five data bases from Jan 1990 to May 2014 with no language restrictions. We screened the 4,464 citations retrieved against exclusion criteria and classified 193 selected references in 12 thematic folders with the aid of the reference management software ENDNOTE X6. After reviewing the full text of all papers we extracted relevant data systematically into a table template to facilitate the synthesising process.

**Results:**

Most studies focused on a particular disease, leaving unexplored the interlinkages between different health conditions and how these relate to legislative, health services, environmental, occupational, and other health determinants. We elucidated some consistent results but there were many heterogeneous findings and several popular beliefs were not fully supported by empirical evidence. Few studies adopted a trans-national perspective and many consisted of cross-sectional descriptions that considered “Hispano-Americans” as a homogeneous category, limiting our analysis. Our results are also constrained by the availability and varying quality of studies reviewed.

**Conclusions:**

Burgeoning research has produced some consistent findings but there are huge gaps in knowledge. To prevent unhelpful generalisations we need a more holistic and nuanced understanding of how mobility, ethnicity, income, gender, legislative status, employment status, working conditions, neighbourhood characteristics and social status intersect with demographic variables and policy contexts to influence the health of the diverse Hispano-American populations present in Europe.

**Electronic supplementary material:**

The online version of this article (doi:10.1186/s12889-015-1799-x) contains supplementary material, which is available to authorized users.

## Background

The last fifteen years have seen a dramatic increase in the population of Hispano-American (HA) origin residing in Europe, with numbers estimated at 2.7 million according to EUROSTAT data in 2011. Spain, in particular, experienced massive migration from South-American countries between 2000 and 2007, where approximately 1,850,000 Hispano-Americans (HAs) registered as new citizens in the National Statistics System (INE).

More recently, the economic recession and high unemployment rates in South-European countries have caused secondary migration flows predominantly directed back to the countries of origin (Additional file 1), but also to the United States and other European countries including the UK, Germany, France, The Netherlands and Switzerland (Additional file 2). In London for example, the HA population has recently increased four-fold placing the UK amongst the European countries with the largest HA population outside of the Mediterranean area [1]. In Switzerland, the majority of undocumented migrants residing in Geneva [2] and Lausanne [3] originate from HA countries.

This relatively new migrant group is composed mainly of persons born in Ecuador, Colombia, Bolivia, Peru and Argentina, who migrate for economic reasons and are employed in low-skilled sectors including services, construction, domestic work and personal care. In Spain, the Hispano-American population has benefited from a “positive selection” legislation including facilities to obtain the Spanish nationality after two years of continued residence in Spain, as opposed to the 10-year requested for migrants of other origins. The Treaty on the Functioning of the European Union (Article 45) grants these new European citizens with full rights to look for a job in another EU country, work there without needing a work permit, residing there for that purpose, staying even after employment has finished, and enjoy equal treatment as EU-born populations in access to employment, working conditions and all other social and tax advantages. With an estimated 583,800 HAs having obtained a Spanish passport between 2004 and 2012 and persistently high unemployment rates in this country, a continuing increase of secondary migratory flows of these new European citizens within the EU is a plausible scenario.

Given that HA immigration to Europe is a relatively new phenomenon, little is known about the health status and its determinants among this population. The extended body of literature on “Latinos’ health” in North-America is of limited applicability to the European context due to divergent legislative frameworks and migrant population profiles.

With the growing dispersion of HAs beyond Mediterranean Europe, there is an increasing need to further our understanding of their most compelling health needs. A broad perspective is needed to account for such needs, transcending the traditional narrow focus on individuals’ health status and “risk behaviours” to account more comprehensively for the various layers of determinants that affect populations’ health, from the individual level to the immediate social environment and the broader social and economic structures of our societies, so “the causes of the causes” are duly accounted for [4].

As part of a larger project financed by the European Commission 7^th^ Framework Programme (COHEMI- *Coordinating Resources to Assess and Improve the Health of Hispano American Migrants in Europe*) this scoping review provides an up-to-date and comprehensive synthesis of the state of the art on this matter in order to inform a research agenda. Drawing on contributions from the social and medical sciences, demography, epidemiology, psychology, psychiatry and economy, we identify current gaps in knowledge and propose specific avenues for future research.

## Methods

We searched systematically the electronic databases EMBASE, GLOBAL HEALTH, MEDLINE/PubMed and SOCIAL POLICY AND PRACTICE using the platform OVID and employing a combination of terms that covered the concepts “Migrant” and “Hispano American”. We adjusted the search strategy iteratively through various scoping searches before we produced a final version. We searched papers published since January 1990 and up to May 2014, with no language restrictions. We also conducted a systematic search through the regional Latin-American database LILACS (“Literatura Latino-Americana y del Caribe en Ciencias de la Salud”) (Table 1). Book chapters and unpublished documents (“grey literature”) were identified by hand-searching citations in the papers reviewed and through web pages of institutions dealing with migrants’ health. This documentation was not reviewed systematically but informed the discussion of our findings.

**Table 1:** **Search strategy**

	**Concept and search fields**	
**Data base**	**Migrant**	**Hispano-American**
Embase 1980–2014 (Week 20)	inmigr*,immigr*,emigr*, migrant*	central america*, south america*, southamerica*, latin america*, latinamerica*,hispano america*,hispanoamerica*, ibero america*, iberoamerica*,iberian america*, hispanic america*, andean* argentin*, bolivia*, chile*, colombia*,costa rica*, costarica*, cuba*, ecuador*, equador*, salvador*, guatemala*, hondura*, mexic*, nicaragua*, panama*, paraguay*, peru*, puerto ric*, puertoric*, dominican*, uruguay*, venezuel*
Global Health 1910–2014 (Week 19)	*Search fields: subject heading,title*	
Ovid MEDLINE (R) 1946- May 2014 (Week 2)		
Social Policy and Practice, 2014-04		
		*Search fields:subject heading, title,abstract*
LILACS	migracion$, migrante$, inmigra$, emigra$	
	*Search fields: key word*	

*/$ = truncation symbols used as substitutes for any string of zero or more characters in the search terms.

**Figure 1:**
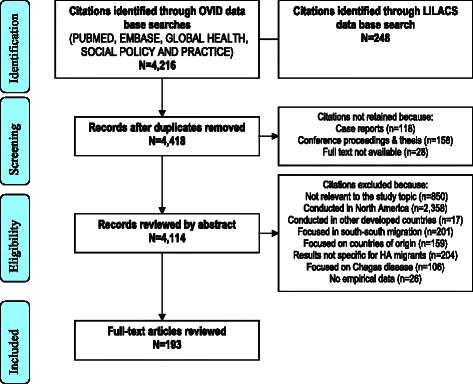
PRISMA flow chart of citations.

The OVID search yielded 4,216 citations (Additional file 3) and we identified 248 additional references through LILACS. We excluded duplicated studies, conference proceedings, studies not conducted in Europe, and those that did not present specific findings related to HAs in their abstract. We also excluded studies focused on Tripanosoma Cruzi/Chagas disease, which is highly prevalent in some HA populations [5], because at least 9 previous systematic reviews have already examined this topic [6-14]. Taking into account the conceptual difficulties related to grouping HA migrants as an analytical category and the conundrums of defining who is a “migrant” [15] we pragmatically considered “HA migrants in Europe” as persons born in any Spanish-speaking country situated in Central-South America/Caribbean, who are currently residing or have ever resided in any European country. The complete citation screening process was conducted by the first author (MR). A second researcher (JML) screened independently 5% of the references retrieved and a 99% agreement resulted from this exercise. The process for selecting studies is described in Figure 1.

All the citations retrieved through OVID were exported into the reference management software ENDNOTE X6. After applying exclusion criteria, a total of 193 references were retained for review. Guided by socio-ecological frameworks [16] we classified citations into 12 thematic folders to account for the different factors that influence individuals’ health, from the individual level to health services and broader contextual factors. We read the full text of all the studies selected and extracted data systematically using a template that included information on the study site, design, study population, sample size and key findings. The tables resulting from this process are included as additional files (Additional file 4, 5, 6, 7, 8, 9, 10, 11, 12, 13, 14 and 15). The PRISMA check list form is provided as Additional file 16.

## Results

Of the 193 studies reviewed, most focused on the health status of HAs, including communicable diseases (38%), non-communicable diseases (17%), maternal and child health (14%), and emotional health (10%). Only a minority addressed health determinants including health service factors (8%), health behaviours (6%) and broader structural and socio-economic factors, including the economic and working environment as well as legislative frameworks and other social determinants of health (7%). (Additional file 17).

Most studies were conducted in Spain (65%), followed by Sweden (13%) and Italy (8%), with some additional papers conducted in other European countries, including Switzerland (3%), the United Kingdom (2%), The Netherlands (1%), Norway (1%), France (1%) and Finland (1%). Studies conducted in countries other than Spain focused mostly on non-communicable diseases, accounting for more than 50% of the studies in this area. Conversely, amongst the studies addressing communicable infections, less than 1/4 were conducted out of Spain (Additional file 18).

The majority of studies consisted of quantitative cross-sectional descriptions and/or analyses, and were conducted after 2008. Only 10 employed qualitative tools (5%) and four adopted a mixed-methods approach (2%). Qualitative research addressed health care services, occupational factors and emotional health, but was almost non-existent in the literature addressing communicable and non-communicable conditions. Only three studies collected data at both country of origin (CO) and destination, truly incorporating a transnational dimension.

Most studies compared specific health indicators of HAs with those of the locally born populations. The “HA population” was often grouped as an analytical category and only a minority of studies focused on nationals from a single country. The country of origin of study participants - sometimes not indicated in the description of the sample - was often not considered in the interpretation of results.

### Health status

- Communicable infections

Of all the studies reviewed 74 (38%) addressed one or more communicable infections. Of these, 29 focused on HIV (Additional file 4), 11 on other sexually transmitted infections (STIs) (Additional file 5), and 14 on Tuberculosis (TB) (Additional file 6). An additional 20 studies focused on other communicable diseases (Additional file 7). With the exception of research focused on a range of “imported diseases” and studies on STIs and TB where the interlinks with HIV were often made explicit, most studies addressed a single specific disease, with limited accounts of the concurrence of different pathologies and how these intersect with individuals’ quality of life.

Studies in Britain [17], Italy [18] and Spain [19] reported higher HIV prevalence rates in homosexual and to a lesser extent heterosexual HA men vs locals, with rates going from 4.5% in heterosexual HA men in Spain [19] to 78% in men who have sex with men (MSM) who injected drugs and attended STI clinics in Italy [20]. In Spain, the most common area of origin of reported HIV cases in the foreign born was HA [21] and the proportion of HIV cases of HA origin increased from 17% to 22% between 2000 and 2007 [22]. A quarter of HIV+ HAs attended at a tropical medicine unit in Spain had not started treatment when indicated [23].

Amongst sex workers, HIV rates differed markedly by sex. A 0.2% prevalence was found in HA female sex workers in Spain [24] while amongst transsexual women HIV rates were above 16% in both Italy and Spain [25-28]. The HIV prevalence in HA male sex workers in Spain (16%) was well above the rates in locals (9%) [29].

Misconceptions about HIV were common amongst HAs in Spain [30,31], where HA MSM reported unprotected anal sex more frequently than local MSM [32]. In the Netherlands, sex workers who originated from HA used condoms less consistently than those originating from other countries and the locally born [33].

A study conducted in England and Wales, reported that 39% of the HIV+ cases identified in persons born in South/Central America had probably acquired the infection in the UK [34]. Similarly, many of the HIV infections in HAs living in Spain appeared to have been locally acquired [35] with the highest percentages of recent infection reported in this population (28%) as compared to locals (23%) and persons from Sub-Saharan Africa (SSA) (12%) [36]. In the European Union (EU), the percentage of Latin-Americans amongst AIDS reports in MSM is increasing [37].

This review also identified some encouraging findings: no differences in response to HIV treatment were found in HAs *vs* locals [38] nor signs of increased transmission of drug resistant HIV [39] and the number of HIV diagnoses in HA children were reported to decrease [40,41]. In Britain, self-reported HIV test uptake reached 90% in MSM born in South/Central America [17] and in Spain, fear of discrimination was not reported to be a major barrier to HIV testing at mobile units [42].

An additional 11 studies focused on other STIs, including the human T-cell lymphotropic virus (HTLV) [43-47], human papilloma virus (HPV) [48-50], syphilis [51], chlamydia [52] and Karposi sarcoma [53]. In Switzerland and Spain, HAs were disproportionally affected by syphilis [51,54]. The prevalence of chlamydia was three times higher in a sample of undocumented migrants (78% originating from Latin-America) who presented at a Swiss hospital to undergo voluntary termination of pregnancy *vs* a control sample of pregnant women with legal residency permit who attended the same hospital during the same time period [52]. The higher prevalence of HTLV-1/2 in HA females *vs* locals [46] and other migrant groups [44,45] prompted suggestions for HTLV antenatal screening in HA pregnant women in Spain [44,45]. Similarly, the high prevalence of HPV infection (21%-62%) [48-50] and increased risk of cervical cancer [55,56] led researchers to highlight the importance of promoting cervical cancer screening in this population [55-57].

We identified 14 studies focused on TB [58-71]. Several studies found high percentages of HAs amongst TB cases diagnosed in migrants [58-63], ranging from 43-44% in migrant children in Spain and Italy [58,61] to 68% in undocumented migrants in Switzerland [62]. In Italy and Switzerland HAs were at higher risk of TB than migrants from other areas [65,66]. In Spain, HAs were more likely to develop TB as an AIDS defining disease than locals [67] and HA inmates were at higher risk of latent TB than Spanish-born prisoners [68].

Only 2.8% of foreign-born TB cases were ill on arrival to Spain but half developed the disease within the following 2 years [59,60]. Although this was often attributed to reactivation of latent infection [60,66] transmission between local and foreign-born communities made an important contribution to the burden of TB [63]. The bidirectional transmission operating between communities was partially attributed to cultural and linguistic “similarities” that enhanced social interaction between HAs and the locally-born [60,63]. The high mobility of this population- including cases of deportation in undocumented migrants- could impede TB treatment compliance [62] although one study found that HAs adhered to treatment better than locals (82% *vs* 76%) [70]. In consistency with studies conducted with other migrant groups [72], misconceptions about TB transmission routes prevailed [71].

An additional 20 studies focused on other communicable diseases. These mainly described the prevalence of cysticercosis [73-75], hepatitis A, B, C and G [76-80], malaria [81], toxoplasma gondii [82,83], intestinal parasitosis, visceral toxocarlasis [84,85], rubella [86,87], varicella [88], and other infectious/parasitic diseases [84,85,89,90]. These studies often drew screening [78-80,87,88] and vaccination recommendations [91,92].

b) Non-communicable conditions

Less attention has been paid to non-communicable conditions, which accounted for 17% of the articles reviewed. We identified 15 studies that focused on cardiovascular diseases (CVD) and related risk factors (Additional file 8), 13 cancer (Additional file 9), and four on other non-communicable conditions (Additional file 10).

Studies on cancer focused on prevalence rates in migrants *vs* non-migrants [56,57,93-99], uptake of gynaecological screening [55,100,101] and psychosocial vulnerabilities in affected children [102]. Prevalence of cancer in HAs *vs* locals varied depending on the type of cancer [98]. HAs were at increased risk of gallbladder [94], testicular [93,98], stomach [98] and papillary thyroid cancer (females) [96], but at lower risk of nervous system [93,97,98], breast [95], ovarian [99], colon cancer [98], and non-Hodgkin’s lymphoma [98]. Risks of cervical cancer increased in HA women aged > 50 [56] and Central Americans [55]. Some studies suggest that genetic and childhood environmental risk factors might explain differences in nervous system, breast, gallbladder and thyroid cancer rates better than exposures after migration [94-97]. Other factors could also influence some types of cancers as illustrated by the lower mammogram uptake reported in HAs *vs* locally born females [100,101].

CVD risks were reported to be lower in HAs *vs* locals in one study [103] but could increase over the years [104]. Important heterogeneities were identified: South Americans in Spain had a reduced risk of mortality from ischemic heart disease [105] but persons originating from Central America and the Caribbean showed the highest mortality rates due to cerebro-vascular pathologies [105-107]. In Sweden, the risk of stroke in Chileans and locals was similar [108].

The prevalence of diabetes mellitus (DM) in HAs was below the level identified in locals and other migrants in a study conducted in a Spanish primary care centre [109]. However, the prevalence of risk factors underlying metabolic conditions linked to diabetes such as obesity, overweight and high body mass index (BMI) was consistently above the level found in autochthonous populations [110-113]. Obesity rates reached 17% in HA males living in Spain [110] and in Sweden the difference in BMI persisted after adjusting for age, educational status, physical activity and smoking [113]. The prevalence of overweight in adopted children of HA origin living in Sweden was particularly high in Chileans (28.6%) as compared with locals (14%), leading researchers to suggest that genetic factors could play a major role in the development of this condition [112]. The role of genetic *vs* environmental exposures was seen as less clear in the case of DM [114]. In one study conducted in Sweden, adoptees and second generation migrants originating from HA had a lower risk for DM than locals [115]. In another study, also conducted in Sweden, the low risk for DM persisted in adoptees from low prevalence South American countries, but increased in children born in Sweden whose parents originated from South America. These findings point to exposures in utero or early childhood as relevant factors to be considered [116].

While non communicable conditions such as osteoarthritis and skin problems tended to be less prevalent amongst HAs there were some exceptions such as headaches, migraines, anaemia, low back pain and constipation [117]. The three studies focused on allergies found that the HA population was disproportionally affected by allergies/asthma [118-120] with symptoms appearing mostly after arrival in Europe [119].

c) Emotional and psychological health

We identified 20 studies (10%) that focused on “emotional and psychological health” (Additional file 11). The presence of vulnerability factors in the HA population living in Europe is well described in the literature and includes exposure to traumatic experiences of violence at the countries of origin [121] as well as post-migration stressors including adaptation to a new context [122-125], cultural barriers [126], economic difficulties [121], inability to meet pre-migration expectations [127], occupational worries [128], poor sense of control [121], break-up of social and family ties, feelings of loss, novelty and nostalgia, undocumented residence status [122], and lack of a consolidated community [129]. In Italy, higher rates of “somatisation” were described amongst HAs [130,131]. However, evidence on the actual prevalence of psychological disorders in this population is inconclusive with studies reporting higher [129,132,133], similar [134], and lower rates [135] *vs* local populations. Differing patterns have also been found when disaggregating the analysis by sex [136] and country of birth [137], including reports of wide disparities in antidepressant consumption [138].

Some studies question the widespread concept of migration as a “risk factor” for poor mental health and suggest that general socio-economic stressors- as opposed to stressors specific to the migration process- could have the greatest impact on mental distress in the HA population. A study in Barcelona found for example that “homesickness” and perceived discrimination were not associated to psychopathology [127] and in Sweden, economic difficulties were stronger risk factors for psychological distress than pre-migration exposure to violence [121]. We identified inconsistencies regarding the role played by perceived discrimination on HA’s health with studies finding both strong [139-141] and no association [134] with mental health indicators.

The use of coping strategies to manage stress was seldom investigated. Religiosity was reported to be high and negatively related to stress in both sexes [142]. Logical analysis, reappraisal, seeking guidance and problem-solving were strategies more widely used than negative coping modalities such as cognitive avoidance, acceptance-resignation, seeking alternative rewards and emotional discharge [128]. However, somatisation, anxiety, hostility and obsessive-compulsive disorders were reported to increase with length of stay, as did the use of avoidance coping strategies [128]. In a study conducted in Spain, social integration in the community positively correlated with subjective well being [123]. However, other studies conducted in Spain found that participation in a community group was positively related to poor mental health [139] and that Ecuadorians from high ethnic density neighbourhoods were more likely to have mental health problems than those living in areas with low ethnic density [143].

d) Maternal and child health

A total of 27 studies addressed maternal and child health [144-170] (Additional file 12). The use of caesarean section (CS) was consistently higher in HAs *vs* locals [146,149-153,160,166] and all the studies that investigated rates of low birth weight (LBW) except for two [151,165] found that these were higher [144,158-161,166] or similar [153] in children born to local women. However, in Finland, newborns to Latin-American mothers had more interventions after birth and higher perinatal mortality [151] and in Sweden, the risk of non-normal birth was 50% higher [163]. In Spain, the risk of some infections [158,164], poor prenatal care [158] and rates of very low birth weight were also higher in children born to HAs mothers when compared with locals [161].

The single study that employed a qualitative approach identified that some HA women in Spain perceived pre-natal controls to offer little benefit [145] although in Sweden, HA women did not use antenatal care either less nor later than recommended [155]. The use of intrapartum epidural analgesia was common [147,150] and mothers were satisfied with its use as a pain reliever [148].

Some researchers pointed to greater use of contraceptive methods in HAs as compared to people from SSA and Asia [168] but there was a strikingly high variability in reported use by country of origin, ranging from 100% in Colombians to 55% in Dominicans [169]. A study with undocumented Latin-American women in Geneva found that four out of five pregnancies resulting in live births were unintended [170].

### Health services

We reviewed 15 studies focused on patterns of service use [171-178], reasons for consultation [179,180], barriers and facilitators to access [181], users’ perceptions over the quality of services [182,183], health providers’ views [184], and the degree of implementation of clinical protocols [185] (Additional file 13).

A study on causes of hospitalization conducted in Spain found that most cases in HAs fell in the “pregnancy and childbirth” category (44%), followed by digestive problems (11%) and trauma (7%). Infectious diseases accounted for 3.2% cases and were the 6th most common cause of admission [179]. Amongst undocumented migrants, primary consultations other than gynaecological and paediatric services were mostly due to acute respiratory infections, depressive disorders, headache, back pain, and hypertension [180].

Access to health services was perceived to be generally easy once a personal health-care card had been obtained. Still, fears of asking for time off at work and mistrust in health providers were found to deter some persons from seeking care in Spain [181]. The perceived quality of services compared favourably to those received at the countries of origin and gratuity was appreciated [178,183]. However, middle-aged Chilean women who had lived in Sweden for 15–20 years highlighted the importance of improving providers’ communication skills in a context of persisting power imbalances between health providers and users [182]. Spanish providers perceived that HAs presented “fewer difficulties” than other immigrants [184] and better compliance with paediatric visits [185].

The use of dermatology [175,176], dental [177] and other specialist services [172] was lower in HAs *vs* locals and an economic analysis found that the health “expenditure” generated by this population was below the local average [174]. Findings regarding the use of emergency services in Spain show some inconsistent results with reports of HA men resorting to emergency services more [172] and less [174] frequently than locals. Still, qualitative inquiries suggest that emergency services are preferred for practical reasons [183] and may be used for non-urgent conditions by HAs who are not entitled to see a family physician [178].

### Social, economic and occupational factors

Only 7% of the studies reviewed focused on social, economic and occupational factors [137,186-198] (Additional file 14).

In Spain, having a monthly income above €1,000 was associated with better self-reported health [193] although one study found that measures of subjective social status predicted heath status better than socio-economic indicators [194]. In Sweden, studies conducted with refugees examined the relationship between migration and self-reported health [195] after adjusting for social class and low material standards. The authors concluded that “being an immigrant” was a risk factor for ill health as important as lifestyle factors [195], and that ethnicity was an independent powerful determinant of self-rated health [196]. In Spain, discrimination emerged as a key factor triggering violence in the HA population [189] and perceived insecurity affected social integration negatively [191]. Studies on self-reported health by sex showed inconclusive results: HA men had better outcomes than HA females in two studies [192,193] but Bolivian men scored the lowest in another [137].

A qualitative exploration with Colombians in Barcelona found that participants were poorly informed about how to prevent labour-related risk hazards [186]. In the UK, a transnational study found that the use of home remedies for minor ailments persisted after migration. Social interactions led to the incorporation of other herbs commonly used by migrants originating from other countries [188]. Reported self-medication with anti-inflammatory drugs, analgesics and antibiotics was high (77%) [198].

### Health behaviours

A total of 11 studies (6%) focused on health behaviours. These addressed mostly dietary habits [199-202] and substance use patterns [203-209] (Additional file 15).

Major dietary changes occurred frequently after migration, including decreased consumption of fruits and soups [200,201] and increased use of processed products [200]. The impact of migration on eating practices was influenced by distance from country of origin, legal status allowing more or less return visits at home, price and availability of local foods, strength of social networks [199], level of ethnic identity [202], and busy work schedules restricting time available to cook [201].

An increased consumption of alcohol after migration was reported often in a study conducted in Spain [206] and about 40% of HA respondents declared that they consumed alcohol frequently [203,209]. Around 25%-30% HAs reported smoking, along the lines of consumption levels in Latin-America and Spain [204,206]. Illicit drugs were perceived as “dangerous” by HA youth [209] who also reported lower use than locals [207]. Reported prevalence of illegal substance use was 5%-6% [203,204], with men in the 25–39 age group being the most frequent users and cocaine the substance most commonly used [208].

## Discussion

This scoping review synthesises existing knowledge about the heath status and determinants of HAs in Europe in order to inform a research agenda. The 193 studies reviewed focused mostly on the health status of this population and paid disproportionately little attention to the determinants of health. The majority of studies addressed communicable infections, followed by “maternal and child health” and a range of “psycho-pathologies”. Burgeoning research in the last few years has yielded some specific, consistent findings about the health status and determinants of HAs in Europe (Table 2). These suggest that this population has a tendency to overweight, is prone to deliver babies using Caesarean sections, is more likely to have post-birth complications than locals, is prone to be affected by allergies/asthma, utilises specialised services less than locals, and carries a disproportionate burden of communicable infections in specific sub-populations. However there are many inconsistent findings and some popular perceptions about “Hispano-American migrants” are not fully confirmed by the evidence reviewed (Table 3). Most studies adopted a vertical approach focusing on a particular disease but left unexplored the interlinkages between different pathologies and health conditions and how these relate to occupational, legislative, social and environmental factors. Many considered “HA migrants” as a homogeneous analytical category masking likely heterogeneities. This review describes these important research gaps and suggests specific avenues of inquiry to advance knowledge in this field.

**Table 2:** **Findings specific to the HA migrant population in Europe that were consistently identified in the literature**

**Results consistently found**	**Evidence**
HIV prevalence in MSM and transgender women of HA origin >16%	17,18,19,20,25,26,27,28
Frequent post-migration acquisition of HIV and TB in HA migrants	34,35,36,60,63
HAs amongst TB cases in migrants > 40%	58,59,60,61,62,63
Prevalence of specific STIs in HA migrants > locals	46,48,50
Prevalence of other communicable diseases (Malaria, Toxoplasmosis, Neurocysticercosis etc.) in HA migrants > locals / high % of HAs amongst total cases diagnosed	73,74,75,81,82,83,90
Prevalence of obesity/overweight/high BMI > locals	110,111,112,113
Prevalence of allergies in HA migrants > locals/other migrant groups	118,119,120
Caesarean Section in HAs > locals	146,149,150,151,152,153,160,166
Some adverse peri-natal outcomes in HAs > locals	151,158,161,163
Use of specialised health services in HAs < locals	172,175,176,177,

**Table 3:** **Popular beliefs not fully supported by the available evidence**

**Popular assumptions**	**Study results**	**Comments**
HA migrants are more prone to psychological disorders than locals	YES:129,132,133	Studies report higher [129,132,133], similar [134] and lower rates [135]. Differing patterns when disaggregating the analysis by sex [136] and country of birth [137]
	NO:134,135	
	Depends on sex and nationality:136,137	
Communicable infections are the main health problem affecting HA migrants	NO:179,180	Most causes of hospitalization fell in the “Pregnancy and Childbirth” category (44%), followed by digestive problems (11%) and trauma (7%). Infectious diseases accounted for 3.2% cases [179]. Main causes of consultations in undocumented migrants: gynaecological and paediatric, acute respiratory infections, depressive disorders, headache, lower back pain and hypertension [180]
HIV and TB are mainly “imported” from countries of origin	NO:34,35,36,60,63	Many HIV cases seem locally acquired [34-36].
		Only 2.8% of foreign-born TB cases ill on arrival but 50% develop disease in 2 years [60]. Reactivation of latent infection [60] but also transmission between local and foreign-born [63]
HAs overuse health services causing operational and financial burdens	Wide heterogeneities: 171,172,174,217	Health “expenditure” HAs < locals [174]. Studies report that HA men resort to emergency services more [172] and less [174] than locals. Wide heterogeneities depending on demographics, health status, area of residence, country of origin and type of service [171,172,174,217]
Strong social networks correlate with better health indicators in HA migrants	YES:123	Social integration in the community positively correlates with subjective well being [123]. Social integration through community group positively related to poor mental health [139]. Ecuadorians from high ethnic density areas more likely to be a possible psychiatric case vs those living in low ethnic density neighbourhoods [143]
	NO:139,143	
Women are the more disadvantaged in terms of poor health	YES:192,193	Heterogeneous results on self-perceived health by sex: men had better outcomes in two studies [192,193] but Bolivian men scored lowest in another [137]
	NOT ALWAYS:137	

It is now widely accepted that the conditions in which people are born, grow, live and work are crucial determinants of populations’ health [210] and amongst “ethnicity and health” scholars there is a high level of consensus on the need to move beyond an exclusive focus on behavioural and genetic determinants to account for broader contextual factors [211]. Still, this review illustrates that the greatest share of scientific production on the health of HAs in Europe is focused on the health status of this population and pays disproportionately little attention to the social, occupational and economic determinants of health. Research efforts have focused on communicable infections although these contribute only partially to the burden of disease in a population that is more commonly affected by gynaecological and obstetric conditions, traumatic accidents, headaches, back pain, depression and gastrointestinal disorders [179,180] (Table 3). This is in line with the wider body of literature addressing migrants’ and ethnic minorities’ health, where these tend to be portrayed as “sources of infection” in spite of being more commonly affected by similar diseases as locals [212,213].

A comprehensive understanding of HAs’ health requires a closer look at various layers of social determinants of health (SDH). We need to understand better the pathways through which the living and working conditions of HA populations in Europe influence their health status and behaviours. Beyond basic demographic characteristics and income indicators we need to account for neighbourhood characteristics such as ethnic density and social cohesion indicators, and consider legal status, religion, social class, employment status, working conditions and ethnicity as cross-cutting axis linking “migrant status” to health outcomes. The specificities of diverse HA populations should be examined, and greater policy attention paid to the “invisibility” of domestic and personal care work - commonly performed by HAs in Europe- and the “unseen” hazards related to it [214]. A careful evaluation of the achievements and pitfalls of policies designed to regularise domestic work, such as the voucher system in Belgium and France [215] and the recent failed attempt in Spain [216] could shed light into what should be done to address this issue.

The literature on migrants’ health reports wide heterogeneities in patterns of health service utilisation, depending on demographic factors, area of residence, country of origin and type of service [171,172,174,217-220]. In the case of HAs, the popular perception that they resort to health care services more often than locals- which is sometimes attributed to their “low tolerance to pain” [221]- is not fully confirmed by the existing evidence (Table 3). A more careful examination that accounts for a variety of demographic and socio-economic factors is needed to unpack the complex patterns and determinants of service use in diverse HA populations.

Cancer risks varied by area of origin and cancer typology [98]. We also identified substantial heterogeneities in cardiovascular mortality [105,107], morbidity [108] and risk factors [104,222]. Our findings mirror the inconsistent picture found for other migrant groups and support recent calls to develop “fine-grained monitoring systems” that account for different populations and health conditions [212]. Future research should investigate how rates of CVD in HAs in Europe compare to those of locally-born residents, HAs who never migrated, those residing in different European countries [223], and “second generation” migrants [103]. However, there are many methodological challenges to be faced, particularly in relation to the availability of ethnicity indicators and reliable comparable data [224].

Our findings do not support the popular belief that HAs exhibit higher rates of psychopathology than the locally born. The inconclusiveness of results in this field - with studies finding levels of psychopathology that are both above and below the rates found in locals- casts doubts on the widespread assumption that migration “per se” is a risk factor of mental disorders (Table 3). Along the same lines, previous reviews on the prevalence of mental illness in migrant populations have also found wide disparities [225]. While the vulnerabilities faced by HA migrants in Europe are well described in the literature, we don’t know enough about how these translate into prevalence of psycho-pathology and the specific factors that trigger or hinder the development of mental disorders. Beyond dissecting the relative influence of stressors directly related with the migration process from other more general strains that also affect the local populations, we need to examine the interlinks between different sources of stress including perceived discrimination, legal status, and interpersonal violence, and how these intersect with other factors such as sex, age, income, occupational status, reasons to migrate, sexual orientation, class and ethnicity [226-228].

In a context of rapidly changing population flows exacerbated by the economic recession, it becomes crucial to longitudinally explore patterns and determinants of health in HAs who initially settled in Spain but who decided to re-emigrate back home or moved to other European countries where the “cultural congruity” that has been described and questioned [134,229] as a “protective factor” in Spain, may not operate. Attention should also be paid to HAs who decide to stay in contexts of high unemployment and job insecurity - firmly established determinants of poor mental health [230-240]. Against the unstable macro-economic backdrop of many European countries, the role played by discrimination as a social determinant of health, and how it interlinks with broader societal attitudes including racism and class prejudices merits additional attention. Further research on the role of social support will help us to interpret the unexpected positive correlations found between poor mental health and indicators of “social integration” [139,143], contributing to current debates over the influence of neighbourhood characteristics on health indicators [241,242].

In spite of unfavourable economic and social contexts, this review has found some promising prospects related to the effective mobilisation of coping strategies in this population [128,142]. The potential benefits (and limitations) of self-esteem and self-efficacy as health-protective factors [243-245] should be further examined and empirical data collected to inform the vulnerability-resilience debate [246].

The inconclusiveness of results in the field of mental health could be partly explained by the difficulty of diagnosing psychiatric disorders in populations of diverse cultural backgrounds with possible differing conceptualisations of mental health and ways to communicate distress [247]. Future studies should consider how psychopathology is defined at the countries of origin [247,248] and investigate further the cultural sensitivity of diagnostic instruments used [225].

Additional research is needed to monitor patterns of substance use and its determinants. We also need to understand better how maternal and child health indicators and determinants vary along the migration experience. The birth weight advantage of children born to HA mother identified in several studies [159,160,166] has led researchers to suggest that a “healthy migrant effect” or selected migration of healthier individuals might operate [159]. We consistently identified poor indicators for other birth outcomes [158,161,163,164] including perinatal mortality [151], which underscores the need to further investigate the nature, extent and duration of the “healthy migrant effect” [249] as well as perceived barriers and facilitators to accessing/providing good quality antenatal care from both users’ and providers’ perspectives. The extremely high rates of unwanted pregnancies [170] reported in the mid 2000s amongst undocumented HA migrants calls for additional up-to-date research in this field.

The majority of studies identified through our search strategy focused on communicable infections with a marked predominance of research focused on Chagas disease. This is not surprising given the particularly high prevalence of this disease amongst HAs, specially Bolivians [5], and the fact that public health policies known to be effective to prevent infection through blood transfusions and from mother-to-child are yet to be adopted by most European countries [250]. Other infectious diseases such as TB and STIs have received less research attention but the available evidence consistently points to a high proportion of HAs amongst TB cases identified in migrants (>40%), with frequent post-migration infection (Table 3). Besides considering how the conditions of migration influence reactivation of latent TB, the extent and mechanisms through which recent, post-migration TB infection occurs within host countries, and how these relate to social networking patterns with the autonomous populations and other migrant groups will further understanding of the nature and extent of the bidirectional transmission patterns reported in the literature [60,63]. Particular focus should also be placed on MSM and transsexual females of HA origin, consistently found to bear a disproportionate burden of HIV. Epidemiological, cost-effectiveness and qualitative studies are needed to assess the value of screening migrants for STIs and other communicable infections. These studies should be framed within broader ethical debates and should consider the fundamental principles of ethnic health research to ensure that besides addressing a public health concern, the evidence generated does actually revert into better health-care experiences and outcomes for minority populations [211].

Most of the studies reviewed adopted a cross-sectional approach based on data collected at a single receiving country with only 3 studies collecting data both at sending and reception countries. The paucity of trans-national and longitudinal studies is partly due to methodological challenges. Further research efforts, coupled with adequate funding should be directed to address these so both pre-migration and post-migration influences can be properly accounted for [251]. Differing reasons for migration should be considered as potential determinants of health and due attention should be paid to the health of migrants who return to their country of origin after a “failed” migration experience.

Amongst the studies that disaggregated data by sex, many found diverging results for women and men, suggesting that sex and gender should be further examined as factors influencing health outcomes in this population. Besides advancing knowledge on the often overlapping vulnerabilities that affect many migrant women, the finding that Bolivian men had some poorer health outcomes than their female counterparts [137] underscores the need to also investigate the reasons behind the health disadvantage described in some HA men. Changes in gender roles and relations resulting from migration processes- and its implications for health- deserve additional research. Moving yet one step further, a broad spectrum of gender identities including those of transgender females should be considered. By accounting for a plurality of “masculinities” and “feminities” future research should contribute to disentangle the often neglected interlink between sex and gender, and how these two variables intersect with other factors, including socioeconomic status and ethnicity, to influence health.

Hispano-Americans share a common language, but they can’t be categorised as a homogeneous “ethnic group”. The wide spectrum of ethnicities across HA populations, very often mixed, is just the most visible of an array of heterogeneities that characterises a population with differing reasons to migrate as well as educational, occupational, and social class backgrounds. Still, many of the studies reviewed grouped together “Latin-Americans” as an analytical category neglecting within-group differences. The diversity of results in the remarkable cases where data was disaggregated by country of origin and sex indicates that more refined analysis are needed so the commonalities and differences *vs* locals, other migrant populations, and within the HA migrant population itself can be better described and understood. To counter the “colour-blind” approach [252] prevailing in the literature, due attention should be paid to “whiteness” [242] and how ethnicity intertwines with migrant and socio-economic status to potentially influence health inequities. The ethnic diversity of the HA population in Europe provides a good opportunity to advance towards a more nuanced understanding of the interlinks between ethnicity, income, migrant status, and social status as determinants of health.

It has been argued that ethnicity and migration have been neglected in the “Social Determinants of Health” agenda [252]. This review illustrates the disproportionately little attention that the social determinants have received in European research addressing Hispano-Americans’ health. We call for continued increasing attention to this field of research, and make specific suggestions on how to readdress the balance. Conceptual models that go beyond categorizing individuals by broad regions of origin and that account for different potential sources of inequities [242,253] should underpin future studies in this field, so over-simplifications and over-generalisations likely to reinforce stigmatising clichés are avoided [252] and the interest of minority groups are better served by health research [211].

### Limitations

The variability of study populations, sampling methods, recruitment strategies and outcome measures used constitute intrinsic limitations of this review. We had to balance the sensitivity *vs* specificity of our search strategy to make the task feasible while at the same time giving an accurate account of the state of the art. We are aware that we might have missed relevant papers and in particular studies on “migrants’ health” that did not include in their abstracts findings that were specific to HAs. However, many of these studies considered “the migrant population” as a homogeneous category or disaggregated their origin in broad terms (E.g.: Latin-American and the Caribbean) tending to mask - rather than shed light- into likely heterogeneities across migrant groups. To minimise potential publication bias we searched the regional database LILACS. We acknowledge that many of the studies that address the health of HAs in Europe are “grey literature” reports that are not published in scientific journals. While not feasible to include these in our review, due attention was paid to relevant documents, which informed our discussion. In spite of the limitations described, we are confident that this review accurately scopes the current state of knowledge on the health status and determinants of HAs in Europe and will be crucial to inform the research agenda on this matter.

## Conclusions

Burgeoning research addressing the health of HAs in Europe has produced some solid findings but there are huge gaps in knowledge and several prevailing beliefs are not fully supported by empirical evidence. Most studies adopted a vertical approach focusing on a particular disease but left unexplored the interlinkages between different pathologies and health conditions and how these relate to the broad determinants of health. Many considered “HA migrants” as a homogeneous analytical category masking likely heterogeneities. To prevent unhelpful generalisations and stereotyping simplifications we need a more holistic and nuanced understanding of how mobility, ethnicity, income, legislative status, employment status, working conditions, neighbourhood characteristics, gender relations and social status intersect with demographic variables and policy contexts to influence the health of the diverse HA populations present in Europe.

## Additional files

Additional file 1:
**Return migration flows of Hispano-Americans initially based in Spain (2008–2013).**
Additional file 2:
**Re-migration flows of Hispano-Americans initially based in Spain (2008–2013).**
Additional file 3:
**Electronic search conducted via OVID on the 22/05/2014.**
Additional file 4:
**Studies on HIV.**
Additional file 5:
**Studies on other STIs.**
Additional file 6:
**Studies on Tuberculosis.**
Additional file 7:
**Studies on other communicable diseases.**
Additional file 8:
**Studies on cardio-vascular diseases and risk factors.**
Additional file 9:
**Studies on cancer.**
Additional file 10:
**Studies on other non-communicable conditions.**
Additional file 11:
**Studies on emotional health.**
Additional file 12:
**Studies on maternal and child health.**
Additional file 13:
**Studies on health services.**
Additional file 14:
**Studies on social, economic and occupational factors.**
Additional file 15:
**Studies on health behaviours.**
Additional file 16:
**PRISMA check list.**
Additional file 17:
**Studies reviewed by topic addressed.**
Additional file 18:
**Studies reviewed by country.**

## Abbreviations

BMI: Body Mass Index
CO: Country of Origin
COHEMI: Coordinating Resources to Assess and Improve the Health of Hispano American Migrants in Europe
CS: Caesarean Section
CVD: Cardiovascular Diseases
DM: Diabetes Mellitus
EU: European Union
HA: Hispano-American
HAs: Hispano-Americans
HIV: Human Immunodeficiency Virus
HPV: Human Papilloma Virus
HTLV: Human T-cell Lymphotropic Virus
LBW: Low Birth Weight
LILACS: Literatura Latino-Americana y del Caribe en Ciencias de la Salud
MSM: Men who have Sex with men
SDH: Social Determinants of Health
SSA: Sub-Saharan Africa
STIs: Sexually Transmitted Infections
TB: Tuberculosis
UK: United Kingdom

## References

[1] Mcllwaine C, Cock JC, Linneker B (2011). No longer invisible: the Latin American community in London.

[2] Commission d’experts “sans-papiers” (2004). Rapport de la commission d’experts pour les travailleurs “sans papiers” à l’intention du Conseil d’Etat Genevois.

[3] Valli M (2004). Les migrants sans permis de séjour à Lausanne.

[4] Marmot M (2005). Social determinants of health inequalities. Lancet.

[5] Roca C, Pinazo MJ, Lopez-Chejade P, Bayo J, Posada E, Lopez-Solana J (2011). Chagas disease among the latin american adult population attending in a primary care center in Barcelona, Spain. PLoS Negl Trop Dis.

[6] Pinazo MJ, Thomas MC, Bua J, Perrone A, Schijman AG, Viotti RJ (2014). Biological markers for evaluating therapeutic efficacy in Chagas disease, a systematic review. Expert Rev Anti-Infect Ther.

[7] Ventura-Garcia L, Roura M, Pell C, Posada E, Gascon J, Aldasoro E (2013). Socio-cultural aspects of Chagas disease: a systematic review of qualitative research. PLoS Negl Trop Dis.

[8] Requena-Mendez A, Lopez MC, Angheben A, Izquierdo L, Ribeiro I, Pinazo MJ (2013). Evaluating Chagas disease progression and cure through blood-derived biomarkers: a systematic review. Expert Rev Anti-Infect Ther.

[9] Afonso AM, Ebell MH, Tarleton RL (2012). A systematic review of high quality diagnostic tests for Chagas disease. PLoS Negl Trop Dis.

[10] Linetzky B, Konfino J, Castellana N, De Maio F, Bahit MC, Orlandini A (2012). Risk of cardiovascular events associated with positive serology for Chagas: a systematic review. Int J Epidemiol.

[11] Abad-Franch F, Vega MC, Rolon MS, Santos WS, Rojas de Arias A (2011). Community participation in Chagas disease vector surveillance: systematic review. PLoS Negl Trop Dis.

[12] Brasil PE, De Castro L, Hasslocher-Moreno AM, Sangenis LH, Braga JU (2010). ELISA versus PCR for diagnosis of chronic Chagas disease: systematic review and meta-analysis. BMC Infect Dis.

[13] Perez-Molina JA, Perez-Ayala A, Moreno S, Fernandez-Gonzalez MC, Zamora J, Lopez-Velez R (2009). Use of benznidazole to treat chronic Chagas’ disease: a systematic review with a meta-analysis. J Antimicrob Chemother.

[14] Bestetti RB, Theodoropoulos TA (2009). A systematic review of studies on heart transplantation for patients with end-stage Chagas’ heart disease. J Card Fail.

[15] Del Amo J (2011). The sexual health of migrants from central and eastern European countries in London: new methods and new data. Sex Transm Infect.

[16] Dahlgren G, Whitehead M (1991). Policies and strategies to promote social equity in health.

[17] Elford J, Doerner R, McKeown E, Nelson S, Anderson J, Low N (2012). HIV infection among ethnic minority and migrant men who have sex with men in britain. Sex Transm Dis.

[18] Giuliani M, Suligoi B (2004). Differences between nonnational and indigenous patients with sexually transmitted infections in Italy and insight into the control of sexually transmitted infections. Sex Transm Dis.

[19] Palacio V, Cuesta MM, Silva D, Varela JA, Lopez C, Vall M (2002). HIV infection among people of foreign origin voluntarily tested in Spain: a comparison with national subjects. Sex Transm Infect.

[20] Suligoi B, Giuliani M, Camisa D, Innocenti M, Nunzi E, Priano L (1997). Sexually transmitted diseases among foreigners in Italy. Epidemiol Infect.

[21] Caro-Murillo AM, Gutierrez F, Manuel Ramos J, Sobrino P, Miro JM, Lopez-Cortes LF (2009). HIV infection in immigrants in Spain: epidemiological characteristics and clinical presentation in the CoRIS Cohort (2004–2006). [Spanish] Infeccion por virus de la inmunodeficiencia humana en inmigrantes en Espana: caracteristicas epidemiologicas y presentacion clinica en la cohorte CoRIS, 2004–2006. Enfermedades Infecciosas y Microbiologia Clinica.

[22] Holguin A, de Mulder M, Yebra G, Lopez M, Soriano V (2008). Increase of non-B subtypes and recombinants among newly diagnosed HIV-1 native spaniards and immigrants in Spain. Curr HIV Res.

[23] Perez-Molina JA, Lopez-Velez R, Navarro M, Perez-Elias MJ, Moreno S (2009). Clinicoepidemiological characteristics of HIV-Infected immigrants attended at a tropical medicine referral unit. J Travel Med.

[24] Belza MJ, Clavo P, Ballesteros J, Menendez B, Castilla J, Sanz S (2004). Social and work conditions, risk behavior and prevalence of sexually transmitted diseases among female immigrant prostitutes in Madrid (Spain). [Spanish] Condiciones sociolaborales, conductas de riesgo y prevalencia de infecciones de transmision sexual en mujeres inmigrantes que ejercen la prostitucion en Madrid. Gaceta Sanitaria / SESPAS.

[25] Gutierrez M, Tajada P, Alvarez A, De Julian R, Baquero M, Soriano V (2004). Prevalence of HIV-1 non-B subtypes, syphilis, HTLV, and hepatitis B and C viruses among immigrant sex workers in Madrid, Spain. J Med Virol.

[26] Spizzichino L, Casella P, Zaccarelli M, Rezza G, Venezia S, Gattari P (1998). HIV infection among foreign people involved in HIV-related risk activities and attending an HIV reference centre in Rome: the possible role of counselling in reducing risk behaviour. AIDS Care.

[27] Spizzichino L, Zaccarelli M, Rezza G, Ippolito G, Antinori A, Gattari P (2001). HIV infection among foreign transsexual sex workers in Rome: prevalence, behavior patterns, and seroconversion rates. Sex Transm Dis.

[28] Zaccarelli M, Spizzichino L, Venezia S, Antinori A, Gattari P (2004). Changes in regular condom use among immigrant transsexuals attending a counselling and testing reference site in central Rome: a 12 year study. Sex Transm Infect.

[29] Belza MJ (2005). Risk of HIV infection among male sex workers in Spain. Sex Transm Infect.

[30] Bermudez MP, Castro A, Buela-Casal G (2011). Psychosocial correlates of condom use and their relationship with worry about STI and HIV in native and immigrant adolescents in Spain. Span J Psychol.

[31] Rios E, Ferrer L, Casabona J, Cayla J, Avecilla A, Gomez IPJ (2009). Knowledge of HIV and sexually-transmitted diseases in Latin American and Maghrebi immigrants in Catalonia (Spain). [Spanish] Conocimiento sobre el VIH y las infecciones de transmision sexual en inmigrantes latinoamericanos y magrebies en Cataluna. Gac Sanit.

[32] Folch C, Munoz R, Zaragoza K, Casabona J (2009). Sexual risk behaviour and its determinants among men who have sex with men in Catalonia, Spain. (Special Issue: HIV/AIDS and other sexually transmitted infections (STI) in men who have sex with men (MSM) - trends and behavioural surveillance). Eurosurveillance.

[33] Van Haastrecht HJ, Fennema JS, Coutinho RA, Van Der Helm TC, Kint JA, Van Den Hoek JA (1993). HIV prevalence and risk behaviour among prostitutes and clients in Amsterdam: migrants at increased risk for HIV infection. Genitourin Med.

[34] Dougan S, Elford J, Sinka K, Fenton KA, Evans BG (2005). Men who have sex with men who are born abroad and diagnosed with HIV in England and Wales: an epidemiological perspective. Int J STD AIDS.

[35] Romero A, Gonzalez V, Esteve A, Martro E, Matas L, Tural C (2012). Identification of recent HIV-1 infection among newly diagnosed cases in Catalonia, Spain (2006–08). Eur J Pub Health.

[36] Romero A, Gonzalez V, Granell M, Matas L, Esteve A, Martro E (2009). Recently acquired HIV infection in Spain (2003–2005): introduction of the serological testing algorithm for recent HIV seroconversion. Sex Transm Infect.

[37] Del Amo J, Likatavicius G, Perez-Cachafeiro S, Hernando V, Gonzalez C, Jarrin I (2011). The epidemiology of HIV and AIDS reports in migrants in the 27 European Union countries, Norway and Iceland: 1999–2006. Eur J Pub Health.

[38] Monge S, Alejos B, Dronda F, Del Romero J, Iribarren J, Pulido F (2013). Inequalities in HIV disease management and progression in migrants from Latin America and sub-Saharan Africa living in Spain. HIV Med.

[39] Yebra G, de Mulder M, Perez-Elias MJ, Perez-Molina JA, Galan JC, Llenas-Garcia J, Moreno S, Holguin A (2011). Increase of transmitted drug resistance among HIV-infected sub-saharan africans residing in Spain in contrast to the native population. PLoS ONE.

[40] Guillen S, Prieto L, Jimenez De Ory S, Gonzalez-Granado I, Gonzalez-Tome MI, Mellado MJ (2012). New diagnosis of HIV infection in children. [Spanish] Nuevos diagnosticos de infeccion VIH en niños. Enferm Infecc Microbiol Clin.

[41] Guillen Martin S, Ramos Amador JT, Resino Garcia R, Bellon Cano JM (2005). Epidemiological trends in new diagnoses of HIV-1 infection in children. [Spanish] Cambios epidemiologicos en nuevos diagnosticos de infeccion por el VIH-1 en niños. Anales de Pediatria.

[42] Hoyos J, Fernandez-Balbuena S, de la Fuente L, Sordo L, Ruiz M, Barrio G (2013). Never tested for HIV in Latin-American migrants and Spaniards: prevalence and perceived barriers. J Int AIDS Soc.

[43] Trevino A, Aguilera A, Caballero E, Benito R, Parra P, Eiros JM, Hernandez MA, Calderon E, Rodriguez-Iglesias M, Torres A (2012). Trends in the prevalence and distribution of HTLV-1 and HTLV-2 infections in Spain. Virol J.

[44] Trevino A, Benito R, Caballero E, Ramos JM, Parra P, Roc L (2011). HTLV infection among foreign pregnant women living in Spain. J Clin Virol.

[45] Ramos JM, Milla A, Trevino A, Sanchez V, Robledano C, Soriano V (2011). Seroprevalence of HTLV infection among immigrant pregnant women in the Mediterranean coast of Spain. J Clin Virol.

[46] Trevino A, Aguilera A, Caballero E, Toro C, Eiros JM, Ortiz de Lejarazu R (2009). Seroprevalence of HTLV-1/2 infection among native and immigrant pregnant women in Spain. AIDS Res Hum Retrovir.

[47] Zehender G, Colasante C, De Maddalena C, Bernini F, Savasi V, Persico T (2004). High prevalence of human T-lymphotropic virus type 1 (HTLV-1) in immigrant male-to-female transsexual sex workers with HIV-1 infection. J Med Virol.

[48] Tornesello ML, Cassese R, de Rosa N, Buonaguro L, Masucci A, Vallefuoco G (2011). High prevalence of human papillomavirus infection in Eastern European and West African women immigrants in South Italy. Apmis.

[49] Del Amo J, Gonzalez C, Losana J, Clavo P, Munoz L, Ballesteros J (2005). Influence of age and geographical origin in the prevalence of high risk human papillomavirus in migrant female sex workers in Spain. Sex Transm Infect.

[50] Gonzalez C, Ortiz M, Canals J, Munoz L, Jarrin I, De La Hera MG (2006). Higher prevalence of human papillomavirus infection in migrant women from Latin America in Spain. Sex Transm Infect.

[51] Gonzalez-Lopez JJ, Fernandez Guerrero ML, Lujan R, Fernandez Tostado S, Gorgolas M, Requena L (2009). Factors determining serologic response to treatment in patients with syphilis. Clin Infect Dis.

[52] Wolff H, Lourenco A, Bodenmann P, Epiney M, Uny M, Andreoli N, Irion O, Gaspoz JM, Dubuisson JB (2008). Chlamydia trachomatis prevalence in undocumented migrants undergoing voluntary termination of pregnancy: a prospective cohort study. BMC Public Health.

[53] De Sanjose S, Marshall V, Sola J, Palacio V, Almirall R, Goedert JJ (2002). Prevalence of Kaposi’s sarcoma-associated herpesvirus infection in sex workers and women from the general population in Spain. Int J Cancer.

[54] Thierfelder C, Weber R, Elzi L, Furrer H, Cavassini M, Calmy A (2012). Participation, characteristics and retention rates of HIV-positive immigrants in the Swiss HIV Cohort Study. HIV Med.

[55] Azerkan F, Zendehdel K, Tillgren P, Faxelid E, Sparen P (2008). Risk of cervical cancer among immigrants by age at immigration and follow-up time in Sweden, from 1968 to 2004. Int J Cancer.

[56] Beiki O, Allebeck P, Nordqvist T, Moradi T (2009). Cervical, endometrial and ovarian cancers among immigrants in Sweden: Importance of age at migration and duration of residence. Eur J Cancer.

[57] Gonzalez Rubio Y, Castano Pinto MS (2003). Descriptive study of cervix cancer screening in our Primary Health Care center: We do not accede the risk population. [Spanish] Estudio descriptivo del cribado del cancer de cervix en nuestro centro de salud. No captamos a la poblacion de riesgo. MEDIFAM Revista de Medicina Familiar y Comunitaria.

[58] Martinez-Roig A, Diz Ardid A, Guevara Carrasco P, Pou Briera I, Ruiz De Larramendi AG, Mombiela Vidal R (2005). Tuberculosis in immigrant children hospitalized in a general hospital in Barcelona, Spain, between January 2000 and December 2005. [Spanish] Estudio de tuberculosis entre niños inmigrantes ingresados en un hospital general de Barcelona durante el periodo de 1 Enero de 2000 a Diciembre de. Acta Pediatrica Espanola 2006.

[59] Arce Arnaez A, Inigo Martinez J, Cabello Ballesteros L, Burgoa Arenales M (2005). Tuberculosis and immigration in a health sanitary area in Madrid, Spain. Trends in 1994–2003. [Spanish] Tuberculosis e inmigracion en un area sanitaria de Madrid. Situacion epidemiologica y evolucion en la decada. Med Clin.

[60] Borrell S, Espanol M, Orcau T, Tudo G, March F, Cayla JA (2010). Tuberculosis transmission patterns among Spanish-born and foreign-born populations in the city of Barcelona. Clin Microbiol Infect.

[61] Giacchino R, Di Martino L, Losurdo G, Pisanti A (2003). Tuberculosis infection and disease in immigrant children. [Italian] Infezione e malattia tubercolare nel bambino immigrato. Infezioni Med.

[62] Perone SA, Bovier P, Pichonnaz C, Rochat T, Loutan L (2005). Tuberculosis in undocumented migrants, Geneva. Emerg Infect Dis.

[63] Rodriguez NA, Chaves F, Inigo J, Bouza E, de Viedma DG, Andres S (2009). Transmission permeability of tuberculosis involving immigrants, revealed by a multicentre analysis of clusters. Clin Microbiol Infect.

[64] Ruiz Lopez FJ, Zarauz Garcia JM, Ortiz Romero MM, Valero Martinez JR, Penalver Mellado C, Sanchez Gascon F, Lorenzo Cruz M (2006). Tuberculosis in the area of Lorca: To adapt or resist. [Spanish]Tuberculosis en la comarca de Lorca: Adaptarse o resistir. Anales de Medicina Interna.

[65] Wolff H, Janssens JP, Bodenmann P, Meynard A, Delhumeau C, Rochat T (2010). Undocumented migrants in Switzerland: geographical origin versus legal status as risk factor for tuberculosis. J immigrant Minority Health / Center Minority Public Health.

[66] Rapiti E, Fano V, Forastiere F, Agabiti N, Geraci S, Scano M (1998). Determinants of tuberculosis in an immigrant population in Rome: a case- control study. Int J Tuberc Lung Dis.

[67] Martin V, de Olalla PG, Orcau A, Cayla JA (2011). Factors associated with tuberculosis as an AIDS-defining disease in an immigration setting. J Epidemiol.

[68] Marco A, Sole N, Orcau A, Escribano M, del Bano L, Quintero S (2012). Prevalence of latent tuberculosis infection in inmates recently incarcerated in a men’s prison in Barcelona. Int J Tuberc Lung Dis.

[69] Ramos JM, Pastor C, Masia Ma M, Cascales E, Royo G, Gutierrez-Rodero F (2003). Health in the immigrant population: prevalence of latent tuberculosis, hepatitis B, hepatitis C, human immunodeficiency virus and syphilis infection. [Spanish] examen de salud en la poblacion inmigrante: prevalencia de infeccion tuberculosa latente, hepatitis B, hepatitis C, infeccion por el VIH y sifilis. Enferm Infecc Microbiol Clin.

[70] Yuguero O, Serna MC, Real J, Galvan L, Riu P, Godoy P (2012). Using treatment compliance to determine the under-notification of tuberculosis in a health region for the years 2007–2009. [Spanish] Cumplimiento terapeutico para determinar la infranotificacion de tuberculosis en una region sanitaria durante los anos 2007–2009. Aten Primaria.

[71] Barbero BS, Hernandez TB (2009). Knowledge, attitudes and perceptions of the Latin-American immigrant population of tuberculosis in the Community of Madrid. [Spanish] Conocimientos, actitudes y percepciones de la poblacion inmigrante latinoamericana enferma de tuberculosis en la Comunidad de Madrid. Aten Primaria.

[72] Abarca Tomas B, Pell C, Bueno Cavanillas A, Guillen Solvas J, Pool R, Roura M (2013). Tuberculosis in migrant populations: a systematic review of the qualitative literature. PLoS One.

[73] Roca C, Gascon J, Font B, Pujol T, Valls ME, Corachan M (2003). Neurocysticercosis and population movements: analysis of 23 imported cases in Spain. Eur J Clin Microbiol Infect Dis.

[74] Terraza S, Pujol T, Gascon J, Corachan M (2001). [Neurocysticercosis: an imported disease?]. Med Clin (Barc).

[75] Zammarchi L, Strohmeyer M, Bartalesi F, Bruno E, Munoz J, Buonfrate D (2013). Epidemiology and management of cysticercosis and Taenia solium taeniasis in Europe, systematic review 1990–2011. PLoS One.

[76] Gergely AE, Bechet S, De Fanti AS, Le Guern AS, Goujon C, Pelicot M (2011). Hepatitis A seroprevalence in a population of immigrants at a french vaccination center. J Travel Med.

[77] Villari P, Ribera G, Nobile CGA, Torre I, Ricciardi G (2001). Antibodies to the E2 protein of GB virus C/hepatitis G virus: prevalence and risk factors in different populations in Italy. Infection.

[78] Valerio L, Barro S, Perez B, Roca C, Fernandez J, Solsona L (2008). Seroprevalence of chronic viral hepatitis markers in 791 recent immigrants in Catalonia, Spain. Screening and vaccination against hepatitis B recommendations. [Spanish]. Seroprevalencia de marcadores de hepatitis cronica virica en 791 inmigrantes recientes en Cataluna, Espana. Recomendaciones de cribado y de vacunacion contra la hepatitis B. Rev Clin Esp.

[79] Masvidal Aliberch RM, Estabanell Buxo A, Miguel Gil B, Cruz Rodriguez C, Frutos Gallego ED, Guzman Molina C (2010). Indication of determination of antibodies against hepatitis C and A viruses in the protocol for the care of young immigrants. [Spanish] Indicacion de la determinacion de los anticuerpos para los virus de la hepatitis C y de la hepatitis A en los protocolos de atencion a los ninos inmigrantes. Gac Sanit.

[80] Bruguera M, Forns X (2006). Hepatitis C in Spain. [Spanish] hepatitis C en Espana. Med Clin.

[81] Baas MC, Wetsteyn JCFM, Van Gool T (2006). Patterns of imported malaria at the academic medical center, Amsterdam, the Netherlands. J Travel Med.

[82] Capretti MG, De Angelis M, Tridapalli E, Orlandi A, Marangoni A, Moroni A (2014). Toxoplasmosis in pregnancy in an area with low seroprevalence: is prenatal screening still worthwhile?. Pediatr Infect Dis J.

[83] Ramos JM, Milla A, Rodriguez JC, Padilla S, Masia M, Gutierrez F (2011). Seroprevalence of Toxoplasma gondii infection among immigrant and native pregnant women in Eastern Spain. Parasitol Res.

[84] Huerga Aramburu H, Lopez-Velez R (2004). Comparative study of infectious diseases in immigrant children from various countries. [Spanish] Estudio comparativo de la patologia infecciosa en ninos inmigrantes de distintas procedencias. Anales de Pediatria.

[85] Turrientes MC, de Ayala AP, Norman F, Navarro M, Perez-Molina J, Rodriquez-Ferrer M (2011). Visceral larva migrans in immigrants from Latin America. Emerg Infect Dis.

[86] Sanz JC, Lemos C, Herrera D, Ramirez-Fernandez R (2004). [Rubella outbreak in a Latin American immigrant population]. Enferm Infecc Microbiol Clin.

[87] Ramos JM, Milla A, Rodriguez JC, Gutierrez F (2012). Rubella immune status among immigrant and nonimmigrant women in Spain. J Med Virol.

[88] Valerio L, Escriba JM, Fernandez-Vazquez J, Roca C, Milozzi J, Solsona L, Molina I (2009). Biogeographical origin and varicella risk in the adult immigration population in Catalonia, Spain (2004–2006). Euro Surveill.

[89] Lopez-Velez R, Huerga H, Turrientes MC (2003). Infectious diseases in immigrants from the perspective of a tropical medicine referral unit. AmJTrop Med Hyg.

[90] Santiago B, Blazquez D, Lopez G, Sainz T, Munoz M, Alonso T (2012). Serological profile of immigrant pregnant women against HIV, HBV, HCV, rubella, Toxoplasma gondii, Treponema pallidum, and Trypanosoma cruzi. [Spanish] Perfil serologico en gestantes extranjeras frente a VIH, VHB, VHC, virus de la rubeola, Toxoplasma gondii, Treponema pallidum, y Trypanosoma cruzi. Enferm Infecc Microbiol Clin.

[91] Navarro Chumbes GC (2011). Vaccine schedule in new resident physicians from peru: Ramon y Cajal university hospital. [Calendario de vacunacion en los nuevos medicos residentes procedentes de peru: hospital Universitario Ramon y Cajal]. Medicina y Seguridad del Trabajo.

[92] Munoz E, Nebot M, Minguell D, Rovira G (2003). Vaccine coverage in the immigrant population in Barcelona. [Spanish]. Med Clin.

[93] Hemminki K, Li X, Czene K (2002). Cancer risks in first-generation immigrants to Sweden. Int J Cancer.

[94] Hemminki K, Mousavi SM, Brandt A, Ji J, Sundquist J (2010). Liver and gallbladder cancer in immigrants to Sweden. Eur J Cancer.

[95] Hemminki K, Mousavi SM, Sundquist J, Brandt A (2011). Does the breast cancer age at diagnosis differ by ethnicity? a study on immigrants to Sweden. Oncologist.

[96] Mousavi SM, Brandt A, Sundquist J, Hemminki K (2011). Risks of papillary and follicular thyroid cancer among immigrants to Sweden. Int J Cancer.

[97] Mousavi SM, Fallah M, Sundquist J, Hemminki K (2011). Nervous system tumors in adult immigrants to Sweden by subsite and histology. Eur J Neurol.

[98] Mousavi SM, Sundquist J, Hemminki K (2013). Cancer incidence among Turkish, Chilean, and North African first-generation immigrants in Sweden compared with residents in the countries of origin and native Swedes. Eur J Cancer Prev.

[99] Mousavi SM, Sundquist K, Hemminki K (2012). Morbidity and mortality in gynecological cancers among first- and second-generation immigrants in Sweden. Int J Cancer.

[100] Puigpinos-Riera R, Pons-Vigues M, Serral G, Rodriguez-Arjona MD, Pasarin MI (2012). I have intention to get a mammogram: Stages of adoption for monitoring mammography in women of different social and cultural background. [Spanish]. Tengo intencion de hacerme una mamografia: Estadios de adopcion para realizar control mamografico en mujeres de distinto origen cultural y social. Psicooncologia.

[101] Sanz-Barbero B, Regidor E, Galindo S (2011). Impact of geographic origin on gynecological cancer screening in Spain. [Spanish]. Rev Saude Publica.

[102] Nacif-Gomera ML, Lorenzo-Gonzalez R, Hernandez M, Perez-Martinez A (2013). AMOR II: an effort to eradicate psychosocial barriers induced by immigration phenomenon in children with cancer. J Pediatr Hematol Oncol.

[103] Zoller B, Li X, Sundquist J, Sundquist K (2012). Risk of venous thromboembolism in first- and second-generation immigrants in Sweden. Eur J Int Med.

[104] Lozano Sanchez ML, Leal Hernandez M, Abellan Huerta J, Gomez Jara P, Ortin Ortin EJ, Abellan Aleman J (2013). Cardiovascular risk of immigrants living in Spain according to origin and years of stay. [Spanish] Evolucion del riesgo cardiovascular de los inmigrantes residentes en Espana segun procedencia y anos de estancia. Aten Primaria.

[105] Regidor E, Astasio P, Calle ME, Martinez D, Ortega P, Dominguez V (2009). The association between birthplace in different regions of the world and cardiovascular mortality among residents of Spain. Eur J Epidemiol.

[106] Regidor E, Ronda E, Pascual C, Martinez D, Calle ME, Dominguez V (2009). Mortality from cardiovascular diseases in immigrants residing in Madrid. [Spanish] Arterial hypertension in immigrant patients in Spanish primary health care. Med Clin.

[107] Regidor E, de La Fuente L, Martinez D, Calle ME, Dominguez V (2008). Heterogeneity in cause-specific mortality according to birthplace in immigrant Men residing in madrid, Spain. Ann Epidemiol.

[108] Khan FA, Zia E, Janzon L, Engstrom G (2004). Incidence of stroke and stroke subtypes in Malmo, Sweden, 1990–2000: marked differences between groups defined by birth country. Stroke.

[109] Roca Vilalta M, Castano Perez A, Lopez Moya C, Lopez Olivares M (2006). Diabetes in a primary care center among spaniards and immigrants. [Spanish] diabetes en un centro de salud entre espanoles e inmigrantes. Pharm Pract.

[110] Marin-Guerrero AC, Gutierrez-Fisac JL, Guallar-Castillon P, Banegas Banegas JR, Regidor E, Rodriguez-Artalejo F (2010). Prevalence of obesity in immigrants in Madrid, Spain. [Spanish] prevalencia de obesidad en inmigrantes en Madrid. Med Clin.

[111] Franch-Nadal J, Martinez-Sierra MC, Espelt A, Sagarra-Busquets E, Patitucci-Gomez F, Goday-Arno A (2013). The diabetic immigrant: cardiovascular risk factors and control. contributions of the IDIME study. [Spanish] El diabetico inmigrante: factores de riesgo cardiovascular y su control. Aportaciones del estudio IDIME. Rev Esp Cardiol.

[112] Johansson-Kark M, Rasmussen F, Hjern A (2002). Overweight among international adoptees in Sweden: a population-based study. Acta Paediatrica, Int J Paediatr.

[113] Wandeil PE, Ponzer S, Johansson SE, Sundquist K, Sundquist J (2004). Country of birth and body mass index: a national study of 2,000 immigrants in Sweden. Eur J Epidemiol.

[114] Serrano-Rios M, Goday A, Larrad TM (1999). Migrant populations and the incidence of type 1 diabetes mellitus: an overview of the literature with a focus on the Spanish-heritage countries in latin America. Diabetes Metab Res Rev.

[115] Ji J, Hemminki K, Sundquist J, Sundquist K (2010). Ethnic differences in incidence of type 1 diabetes among second-generation immigrants and adoptees from abroad. J Clin Endocrinol Metab.

[116] Soderstrom U, Aman J, Hjern A (2012). Being born in Sweden increases the risk for type 1 diabetes - a study of migration of children to Sweden as a natural experiment. Acta Paediatr.

[117] Esteban-Vasallo MD, Dominguez-Berjon MF, Astray-Mochales J, Genova-Maleras R, Perez-Sania A, Sanchez-Perruca L (2009). Prevalence of diagnosed chronic disorders in the inmigrant and native population. [Spanish] Prevalencia de enfermedades cronicas diagnosticadas en poblacion inmigrante y autoctona. Gac Sanit.

[118] Burastero SE, Masciulli A, Villa AM (2011). Early onset of allergic rhinitis and asthma in recent extra-european immigrants to Milan, Italy: the perspective of a non-governmental organisation. Allergol Immunopathol.

[119] Tedeschi A, Barcella M, Dal Bo GA, Miadonna A (2003). Onset of allergy and asthma symptoms in extra-european immigrants to Milan, Italy: possible role of environmental factors. Clin Exp Allergy.

[120] Dominguez-Ortega J, Gonzalz de Olano D, Trujillo MJ, Henriquez A, Losada A, Rodriguez-Dominguez B (2011). Allergic sensitization profile in the immigrant population living in the central region of Spain. [Spanish] Perfil de sensibilizacion alergica en inmigrantes residentes en la zona centro de Espana. Anales del Sistema Sanitario de Navarra.

[121] Sundquist J, Bayard-Burfield L, Johansson LM, Johansson SE (2000). Impact of ethnicity, violence and acculturation on displaced migrants: psychological distress and psychosomatic complaints among refugees in Sweden. J Nerv Ment Dis.

[122] Kirchner T, Patino C (2011). Latin-American immigrant women and mental health: differences according to their rural or urban origin. Spanish J Psychol.

[123] Herrero J, Fuente A, Gracia E (2011). Covariates of subjective well-being among Latin American immigrants in Spain: the role of social integration in the community. J Commun Psychol.

[124] Sendra-Gutierrez JM, De Francisco Beltran P, Iribarren M, Vargas Aragon ML (2012). Outpatient psychiatric care in the immigrant population of Segovia (2001–2008): Descriptive study. [Spanish] Asistencia psiquiatrica ambulatoria en la poblacion inmigrante de Segovia (2001–2008): Estudio descriptivo. Revista de Psiquiatria y Salud Mental.

[125] Revollo HW, Qureshi A, Collazos F, Casas M (2011). The impact of social context on the acculturative process: acculturative stress in Latin American immigrants. Euro Psychiatry.

[126] Sundquist J, Iglesias E, Isacsson A (1995). Migration and health:a study of Latin American refugees, their exile in Sweden and repatriation. Scand J Prim Health Care.

[127] Zarza MJ, Sobrino Prados MI (2007). Estrés de adaptación sociocultural en inmigrantes latinoamericanos residentes en Estados Unidos *vs* España: Una revisión bibliográfica. Anales de psicología.

[128] Patino C, Kirchner T (2010). Stress and psychopathology in latin-american immigrants: the role of coping strategies. Psychopathology.

[129] Qureshi A, Collazos F, Sobradiel N, Eiroa-Orosa FJ, Febrel M, Revollo-Escudero HW (2013). Epidemiology of psychiatric morbidity among migrants compared to native born population in Spain: a controlled study. Gen Hosp Psychiatry.

[130] Aragona M, Monteduro MD, Colosimo F, Maisano B, Geraci S (2008). Effect of gender and marital status on somatization symptoms of immigrants from various ethnic groups attending a primary care service. German J Psychiatry.

[131] Aragona M, Rovetta E, Pucci D, Spoto J, Villa AM (2012). Somatization in a primary care service for immigrants. Ethn Health.

[132] Bayard-Burfield L, Sundquist J, Johansson SE (2001). Ethnicity, self reported psychiatric illness, and intake of psychotropic drugs in five ethnic groups in Sweden. J Epidemiol Community Health.

[133] Lipsicas CB, Makinen IH, Apter A, De Leo D, Kerkhof A, Lonnqvist J (2012). Attempted suicide among immigrants in European countries: an international perspective. Soc Psychiatry Psychiatr Epidemiol.

[134] Revollo HW, Qureshi A, Collazos F, Valero S, Casas M (2011). Acculturative stress as a risk factor of depression and anxiety in the Latin American immigrant population. Int Rev Psychiatry.

[135] Pascual JC, Malagon A, Corcoles D, Gines JM, Soler J, Garcia-Ribera C (2008). Immigrants and borderline personality disorder at a psychiatric emergency service. Br J Psychiatry.

[136] Del Amo J, Jarrin I, Garcia-Fulgueiras A, Ibanez-Rojo V, Alvarez D, Rodriguez-Arenas MA (2011). Mental health in Ecuadorian migrants from a population-based survey: the importance of social determinants and gender roles. Soc Psychiatry Psychiatr Epidemiol.

[137] Villarroel N, Artazcoz L (2012). Heterogeneous patterns of health status among immigrants in Spain. Healt Place.

[138] Cruz I, Serna C, Real J, Rue M, Soler J, Galvan L (2010). Comparison of the consumption of antidepressants in the immigrant and native populations in a Spanish health region: an observational study. BMC Public Health.

[139] Llacer A, Del Amo J, Garcia-Fulgueiras A, Ibanez-Rojo V, Garcia-Pino R, Jarrin I (2009). Discrimination and mental health in Ecuadorian immigrants in Spain. J Epidemiol Community Health.

[140] Gonzalez-Castro JL, Ubillos S (2011). Determinants of psychological distress among migrants from Ecuador and Romania in a Spanish city. Int J Soc Psychiatry.

[141] Ruiz Hernandez JA, Torrente Hernandez G, Rodriguez Gonzalez A, Ramirez de la Fe MC (2011). Acculturative stress in Latin-American immigrants: an assessment proposal. Span J Psychol.

[142] Kirchner T, Patino C (2010). Stress and depression in Latin American immigrants: the mediating role of religiosity. Eur Psychiatry.

[143] Jarrin I, Garcia-Fulgueiras A, Ibanez-Rojo V, Alvarez D, Garcia-Pina R, Fernandez-Liria A (2013). Absence of protective ethnic density effect on Ecuadorian migrants’ mental health in a recent migration setting: a multilevel analysis. Soc Psychiatry Psychiatr Epidemiol.

[144] Agudelo-Suarez AA, Ronda-Perez E, Gil-Gonzalez D, Gonzalez-Zapata LI, Regidor E (2009). [Relationship in Spain of the length of the gestation and the birth weight with mother’s nationality during the period 2001–2005]. Rev Esp Salud Publica.

[145] Barona-Vilar C, Mas-Pons R, Fullana-Montoro A, Giner-Monfort J, Grau-Munoz A, Bisbal-Sanz J (2013). Perceptions and experiences of parenthood and maternal health care among Latin American women living in Spain: a qualitative study. Midwifery.

[146] Bernis C, Varea C, Bogin B, Gonzalez-Gonzalez A (2013). Labor management and mode of delivery among migrant and Spanish women: does the variability reflect differences in obstetric decisions according to ethnic origin?. Matern Child Health J.

[147] Ekeus C, Cnattingius S, Hjern A (2010). Epidural analgesia during labor among immigrant women in Sweden. Acta Obstet Gynecol Scand.

[148] Gredilla E, Perez Ferrer A, Martinez B, Alonso E, Diez J, Gilsanz F (2008). [Maternal satisfaction with the quality of epidural analgesia for pain relief in labor]. Rev Esp Anestesiol Reanim.

[149] Hernandez-Rivas E, Flores-Le Roux JA, Benaiges D, Sagarra E, Chillaron JJ, Paya A (2013). Gestational diabetes in a multiethnic population of Spain: clinical characteristics and perinatal outcomes. Diabetes Res Clin Pract.

[150] Jimenez-Puente A, Benitez-Parejo N, Del Diego-Salas J, Rivas-Ruiz F, Maanon-Di Leo C (2012). Ethnic differences in the use of intrapartum epidural analgesia. BMC Health Serv Res.

[151] Malin M, Gissler M (2009). Maternal care and birth outcomes among ethnic minority women in Finland. BMC Public Health.

[152] Merry L, Small R, Blondel B, Gagnon AJ (2013). International migration and caesarean birth: a systematic review and meta-analysis. BMC Pregnancy Childbirth.

[153] Merten S, Wyss C, Ackermann-Liebrich U (2007). Caesarean sections and breastfeeding initiation among migrants in Switzerland. Int J Public Health.

[154] Nedstrand E, Ekseth U, Lindgren R, Hammar M (1995). The climacteric among South-American women who immigrated to Sweden and age-matched Swedish women. Maturitas.

[155] Ny P, Dykes AK, Molin J, Dejin-Karlsson E (2007). Utilisation of antenatal care by country of birth in a multi-ethnic population: a four-year community-based study in Malmo, Sweden. Acta Obstet Gynecol Scand.

[156] Perez-Alcala I, Sievert LL, Obermeyer CM, Reher DS (2013). Cross-cultural analysis of determinants of hot flashes and night sweats: Latin-American immigrants to Madrid and their Spanish neighbors. Menopause.

[157] Pérez-Alcalá I, Sievert LL, Obermeyer CM, Reher DS (2013). Cross cultural analysis of factors associated with age at natural menopause among Latin-American immigrants to Madrid and their Spanish neighbors. Am J Hum Biol.

[158] Puig Sola C, Zarzoso Palomero A, Garcia-Algar O, Cots Reguant F, Buron Pust A, Castells Oliveres X (2008). [Hospital admission in newborns according to ethnicity and parents’ country of origin in an urban area of Barcelona [Spain]]. Gac Sanit.

[159] Restrepo-Mesa SL, Estrada-Restrepo A, Gonzalez-Zapata LI, Agudelo-Suarez AA, Ronda-Perez E (2010). Factors related to birth weight: A comparison of related factors between newborns of Spanish and Colombian immigrant women in Spain. [Spanish] Peso al nacer: una comparacion de sus factores relacionados entre los recien nacidos de madres Espanolas y madres Colombianas residentes en Espana. Arch Latinoam Nutr.

[160] Rio I, Castello A, Barona C, Jane M, Mas R, Rebagliato M (2010). Caesarean section rates in immigrant and native women in Spain: the importance of geographical origin and type of hospital for delivery. Eur J Pub Health.

[161] Rio I, Castello A, Jane M, Prats R, Barona C, Mas R (2010). Reproductive and perinatal health indicators in immigrant and Spanish-born women in Catalonia and Valencia (2005–2006). [Spanish] Indicadores de salud reproductiva y perinatal en mujeres inmigrantes y autoctonas residentes en Cataluna y en la Comunitat Valenciana. Gaceta Sanitaria.

[162] Rio I, Castello-Pastor A, del Val Sandin-Vazquez M, Barona C, Jane M, Mas R (2011). Breastfeeding initiation in immigrant and non-immigrant women in Spain. Eur J Clin Nutr.

[163] Robertson E, Malmstrom M, Johansson SE (2005). Do foreign-born women in Sweden have an increased risk of non-normal childbirth?. Acta Obstet Gynecol Scand.

[164] Sampedro A, Mazuelas P, Rodriguez-Granger J, Torres E, Puertas A, Navarro JM (2010). Serological markers in immigrant and Spanish pregnant women in Granada. [Spanish] Marcadores serologicos en gestantes inmigrantes y autctonas en Granada. Enferm Infecc Microbiol Clin.

[165] Urquia ML, Glazier RH, Blondel B, Zeitlin J, Gissler M, Macfarlane A (2010). International migration and adverse birth outcomes: role of ethnicity, region of origin and destination. J Epidemiol Community Health.

[166] Vangen S, Stoltenberg C, Skrondal A, Magnus P, Stray-Pedersen B (2000). Cesarean section among immigrants in Norway. Acta Obstet Gynecol Scand.

[167] Vikanes A, Grjibovski AM, Vangen S, Magnus P (2008). Length of residence and risk of developing hyperemesis gravidarum among first generation immigrants to Norway. Eur J Pub Health.

[168] Saurina C, Vall-Llosera L, Saez M (2012). Factors determining family planning in Catalonia: sources of inequity. Int J Equity Health.

[169] Hernando V (2005). Conocimientos y uso de anticonceptivos en la poblacion inmigrante latinoamericana en la comunidad autonoma de Madrid. Boletin Epidemiologico Semanal.

[170] Wolff H, Stalder H, Epiney M, Walder A, Irion O, Morabia A (2005). Health care and illegality: a survey of undocumented pregnant immigrants in Geneva. Soc Sci Med.

[171] Saurina C, Vall-Llosera L, Saez M (2012). Factors determining access to and use of primary health care services in the Girona Health Region (Spain). Eur J Health Econ.

[172] Sanz B, Regidor E, Galindo S, Pascual C, Lostao L, Diaz JM (2011). Pattern of health services use by immigrants from different regions of the world residing in Spain. Int J Public Health.

[173] Munoz de Bustillo R, Anton JI (2010). Use of public health services by Latin American immigrants in Spain. [Spanish] Utilizacion de los servicios publicos de salud por parte de la poblacion inmigrante latinoamericana en Espana. Salud Publica Mex.

[174] Lopez Nicolas A, Ramos Parreno JM (2009). Health services utilization by the immigrant and native-born populations in the autonomous region of Murcia (Spain). [Spanish] Utilizacion de servicios sanitarios por parte de las poblaciones inmigrante y nativa en la Comunidad Autonoma de la Region de Murcia. Gac Sanit.

[175] Taberner R, Nadal C, Llambrich A, Vila EI, Torne A (2010). Dermatology service utilization and reasons for consultation by Spanish and immigrant patients in the region served by Hospital Son Llatzer, Palma de Majorca, Spain. [Spanish] Motivos de consulta dermatologicos en la poblacion inmigrante y espanola del area de salud del Hospital Son Llatzer (Mallorca). Actas dermo-sifiliograficas.

[176] Albares Tendero MP, Ramos Rincon JM, Belinchon Romero I, Betlloch Mas I, Pastor Tomas N, Botella Anton R (2008). [Utilization of dermatology services among the immigrant population]. Gac Sanit.

[177] Hjern A, Grindefjord M (2000). Dental health and access to dental care for ethnic minorities in Sweden. Ethn Health.

[178] Brigidi S, Cremonesi P, Cristina ML, Costaguta C, Sartini M (2008). Inequalities and health: analysis of a model for the management of Latin American users of an emergency department. J Prev Med Hyg.

[179] Ortells-Ros E, Navarro-Calderon E, Abad I, Borras R, Carbonell E, Gonzalez A (2011). Hospital admissions among the immigrant population in 2006–10 vs. 2001–02 in the city of Valencia. [Spanish]. Boletin Epidemiologico Semanal.

[180] Esteban y Pena MM (2001). Motives for consultation and demographic characteristics of a community of “undocumented” immigrants in the district of Usera-Villaverde (Madrid). [Spanish] Motivos de consulta y caracteristicas demograficas de una comunidad de inmigrantes “sin papeles” en el distrito de Usera-Villaverde (Madrid). Atencion primaria / Sociedad Espanola de Medicina de Familia y Comunitaria.

[181] Terraza-Nunez R, Toledo D, Vargas I, Vazquez ML (2010). Perception of the Ecuadorian population living in Barcelona regarding access to health services. Int J Public Health.

[182] Binfa L, Robertson E, Ransjo-Arvidson AB (2010). “We are always asked; ‘where are you from?’”: Chilean women’s reflections in midlife about their health and influence of migration to Sweden. Scand J Caring Sci.

[183] Bermúdez E (2004). Imágenes de la salud y enfermedad de las mujeres colombianas inmigrantes en España / Images of the health and disease of the Colombian immigrant women in Spain. Rev Gerenc Políticas Salud.

[184] Fernandez-Castillo A, Vilchez-Lara MJ (2009). Professional opinion about hospitalising latin-american immigrant children in andalucia, Spain. [Spanish] Opinion profesional sobre la hospitalizacion infantil de inmigrantes de origen latinoamericano en andalucia, espana. Revista de Salud Publica.

[185] Recasens Oliva E, Closa R, Tregon J, Juarez MM (2008). Are we implementing the guidelines for the care of immigrant children correctly?[Catalan] Apliquem correctament el protocol del nen immigrant?. Pediatria Catalana.

[186] Agudelo-Suarez A, Ronda-Perez E, Gil-Gonzalez D, Vives-Cases C (2008). A group of Colombian immigrants’ self-perception regarding working conditions and health in Alicante, Spain. [Spanish] Percepcion sobre condiciones de trabajo y salud de la poblacion inmigrante Colombiana en Alicante, Espana. Revista de Salud Publica.

[187] Benitez Robredo T, Llerena Achutegui P, Lopez Gimenez R, Brugera Moreno C, Lasheras Lozano L (2004). Socioeconomic determinants in immigrant families. [Spanish] Determinantes socioeconomicos en un colectivo de familias inmigrantes. Anales de Pediatria.

[188] Ceuterick M, Vandebroek I, Pieroni A (2011). Resilience of Andean urban ethnobotanies: a comparison of medicinal plant use among Bolivian and Peruvian migrants in the United Kingdom and in their countries of origin. J Ethnopharmacol.

[189] Colorado-Yohar S, Tormo MJ, Salmeron D, Dios S, Ballesta M, Navarro C (2012). Violence reported by the immigrant population is high as compared with the native population in Southeast Spain. J Interpersonal Violence.

[190] Dunlavy AC (2013). Health inequalities among workers with a foreign background in Sweden: do working conditions matter?. Int J Environ Res Public Health.

[191] Fuente A, Herrero J (2012). Social integration of Latin-American immigrants in Spain: the influence of the community context. Span J Psychol.

[192] Rodriguez Alvarez E, Lanborena Elordui N, Senhaji M, Perida Riguera C (2008). Sociodemographic variables and lifestyle as predictors of self-perceived health in immigrants in the Basque Country. [Spanish] Variables sociodemograficas y estilos de vida como predictores de la autovaloracion de la salud de los inmigrantes en el Pais Vasco. Gac Sanit.

[193] Salinero-Fort MA, Jimenez-Garcia R, del Otero-Sanz L, de Burgos-Lunar C, Chico-Moraleja RM, Martin-Madrazo C, Gomez-Campelo P (2012). Self-reported health status in primary health care: the influence of immigration and other associated factors. PLoS ONE.

[194] Sanchon-Macias MV (2013). Relationship between subjective social status and perceived health among Latin American immigrant women. Revista Latino-Americana de Enfermagem.

[195] Sundquist J (1995). Living conditions and health: a population-based study of labour migrants and Latin American refugees in Sweden and those who were repatriated. Scand J Prim Health Care.

[196] Sundquist J (1995). Ethnicity, social class and health: a population-based study on the influence of social factors on self-reported illness in 223 Latin American refugees, 333 Finnish and 126 South European labour migrants and 841 Swedish controls. Soc Sci Med.

[197] Wright K (2011). Constructing migrant wellbeing: an exploration of life satisfaction amongst Peruvian migrants in London. J Ethn Migr Stud.

[198] González-López J, Rodríguez-Gázquez M, Lomas-Campos M (2012). Automedicación en inmigrantes latinoamericanos adultos de Sevilla. Acta Paul Enferm.

[199] Codesal DM (2010). Eating abroad, remembering (at) home: three foodscapes of Ecuadorian migration in New York, London and Santander. Anthropol Food.

[200] Gilbert PA, Khokhar S (2008). Changing dietary habits of ethnic groups in Europe and implications for health. Nutr Rev.

[201] Posada E, Pell C, Angulo N, Pinazo MJ, Gimeno F, Elizalde I (2011). Bolivian migrants with Chagas disease in Barcelona, Spain: a qualitative study of dietary changes and digestive problems. Int Health.

[202] Romo R, Gil JM (2012). Ethnic identity and dietary habits among Hispanic immigrants in Spain. Br Food J.

[203] González López JR, Lomas-Campos MM, García Fernández J, Pascualvaca Armario J, Guardado González M, Muñoz Guardado B (2010). Conductas de salud en inmigrantes latinoamericanos adultos del Distrito Macarena de Sevilla (España). Invest Educ Enferm.

[204] Gonzalez-Lopez JR, Rodriguez-Gazquez MA, Lomas-Campos MM (2012). Prevalence of alcohol, tobacco and street drugs consumption in adult Latin American immigrants. Revista Latino-Americana de Enfermagem.

[205] Monras M, Freixa N, Ortega L, Pineda P, Gonzalez A, Gual A (2006). Alcoholism and immigration, adherence of immigrant patients to group therapy. Med Clin.

[206] Tortajada S, Llorens N, Castellano M, Alvarez FJ, Aleixandre-Benavent R, Valderrama-Zurian JC (2010). Perception and consumption of alcohol among the immigrant population from Latin America in Valencia region (Spain). Substance Misuse.

[207] Marsiglia FF, Kulis S, Luengo MA, Nieri T, Villar P (2008). Immigrant advantage? Substance use among Latin American immigrant and native-born youth in Spain. Ethn Health.

[208] Tordable Merino I, Sanchez Sanchez A, Santos Sanz S, Garcia Vicario MI, Redondo Martin S (2010). Trends in drug consumption among immigrants between 2004 and 2008. [Spanish] Evolucion del consumo de drogas por inmigrantes entre los anos 2004 y 2008. Gaceta Sanitaria.

[209] Tortajada Navarro S, Valderrama Zurian JC, Castellano Gomez M, Llorens Aleixandre N, Agullo Calatayud V, Herzog B (2008). Drug consumption and perception among Latin American immigrants [Spanish]. Psicothema.

[210] Marmot M, Allen J, Bell R, Bloomer E, Goldblatt P, Consortium for the European Review of Social Determinants of Health (2012). WHO European review of social determinants of health and the health divide. Lancet.

[211] Mir G, Salway S, Kai J, Karlsen S, Bhopal R, Ellison GT (2013). Principles for research on ethnicity and health: the Leeds Consensus Statement. Eur J Public Health.

[212] Kunst A, Stronks K, Agyemang C, Bernd R, Mladovsky P, Devillé W, Rijks B, Petrova-Benedict R, McKee M (2011). Non-Communicable diseases. Migration and health in the European Union.

[213] Gill PS, Kai J, Bhopal RS, Wild S, Raftery J (2007). Health care needs assessment: black and minority ethnic groups. Health care needs assessment.

[214] Ahonen EQ, Lopez-Jacob MJ, Vazquez ML, Porthe V, Gil-Gonzalez D, Garcia AM (2010). Invisible work, unseen hazards: the health of women immigrant household service workers in Spain. Am J Ind Med.

[215] Michielsen J, Willems R, Nouwen W, Jalhay S (2013). Promoting integration for migrant domestic workers in Belgium-Executive Summary.

[216] Marzo C. Fracasa la ley para regularizar a las empleadas del hogar. El Periódico. 4th July 2012. Available at: http://www.elperiodico.com/es/noticias/sociedad/fracasa-ley-para-regularizar-las-empleadas-del-hogar-2019961.

[217] Regidor E, Sanz B, Pascual C, Lostao L, Sanchez E, Diaz Olalla JM (2009). Health services utilization by the immigrant population in Spain. [Spanish]. Gac Sanit.

[218] Norredam M, Krasnik A (2011). Migrants’ access to health services. Migration and health in the European Union.

[219] Anton JI, Munoz de Bustillo R (2010). Health care utilisation and immigration in Spain. Eur J Health Econ.

[220] Uiters E, Deville W, Foets M, Spreeuwenberg P, Groenewegen PP (2009). Differences between immigrant and non-immigrant groups in the use of primary medical care; a systematic review. BMC Health Serv Res.

[221] Brigidi S, Comelles JM, Allué X, Bernal M, Fernández-Rufete J, Mascarella L (2009). Culturas médicas y emigrantes en el casco antiguo de Génova. Migraciones y Salud.

[222] González-López J, Lomas-Campos M, Rodríguez-Gázquez M (2013). Factores de riesgo y eventos cardiovasculares en inmigrantes latinoamericanos adultos en el Distrito Macarena, Sevilla, España: estudio piloto. Rev Esc Enfermeria USP.

[223] Bhopal RS, Rafnsson SB, Agyemang C, Fagot-Campagna A, Giampaoli S, Hammar N (2012). Mortality from circulatory diseases by specific country of birth across six European countries: test of concept. Eur J Public Health.

[224] Rafnsson SB, Bhopal RS (2009). Large-scale epidemiological data on cardiovascular diseases and diabetes in migrant and ethnic minority groups in Europe. Eur J Public Health.

[225] Lindert J, Schouler-Ocak M, Heinz A, Priebe S (2008). Mental health, health care utilisation of migrants in Europe. Eur Psychiatry.

[226] Borrell C, Muntaner C, Sola J, Artazcoz L, Puigpinos R, Benach J (2008). Immigration and self-reported health status by social class and gender: the importance of material deprivation, work organisation and household labour. J Epidemiol Community Health.

[227] Farmer MM, Ferraro KF (2005). Are racial disparities in health conditional on socioeconomic status?. Soc Sci Med.

[228] Alvarez-del Arco D, del Amo J, Garcia-Pina R, Garcia-Fulgueiras AM, Rodriguez-Arenas MA, Ibanez-Rojo V (2013). Violence in adulthood and mental health: gender and immigrant status. J Interpers Violence.

[229] Rico J, Comelles JM, Allué X, Bernal M, Fernández-Rufete J, Mascarella L (2009). La atención primaria de salud ante el reto de la asistencia en extranjería: los ecuatorianos en un municipio murciano. Migraciones y Salud.

[230] Chen L, Li W, He J, Wu L, Yan Z, Tang W (2012). Mental health, duration of unemployment, and coping strategy: a cross-sectional study of unemployed migrant workers in eastern china during the economic crisis. BMC Public Health.

[231] Limb M (2011). Scale of youth unemployment is a public health emergency, Marmot says. BMJ.

[232] Puig-Barrachina V, Malmusi D, Martenez JM, Benach J (2011). Monitoring social determinants of health inequalities: the impact of unemployment among vulnerable groups. Int J Health Serv.

[233] Lindstrom M (2005). Psychosocial work conditions, unemployment and self-reported psychological health: a population-based study. Occup Med (Lond).

[234] Artazcoz L, Benach J, Borrell C, Cortes I (2004). Unemployment and mental health: understanding the interactions among gender, family roles, and social class. Am J Public Health.

[235] Ferrie JE, Martikainen P, Shipley MJ, Marmot MG, Stansfeld SA, Smith GD (2001). Employment status and health after privatisation in white collar civil servants: prospective cohort study. BMJ.

[236] Cable N, Sacker A, Bartley M (2008). The effect of employment on psychological health in mid-adulthood: findings from the 1970 British Cohort Study. J Epidemiol Community Health.

[237] Blakely TA, Collings SC, Atkinson J (2003). Unemployment and suicide: evidence for a causal association?. J Epidemiol Community Health.

[238] Ferrie JE, Shipley MJ, Stansfeld SA, Marmot MG (2002). Effects of chronic job insecurity and change in job security on self reported health, minor psychiatric morbidity, physiological measures, and health related behaviours in British civil servants: the Whitehall II study. J Epidemiol Community Health.

[239] Montgomery SM, Cook DG, Bartley MJ, Wadsworth ME (1999). Unemployment pre-dates symptoms of depression and anxiety resulting in medical consultation in young men. Int J Epidemiol.

[240] Marmot M, Wilkinson R (2006). Social determinants of health.

[241] Pickett KE, Wilkinson RG (2008). People like us: ethnic group density effects on health. Ethn Health.

[242] Viruell-Fuentes EA, Miranda PY, Abdulrahim S (2012). More than culture: structural racism, intersectionality theory, and immigrant health. Soc Sci Med.

[243] Gentil I (2009). Salud y mujeres inmigrantes latinoamericanas. Autoestima y resiliencia. Index Enferm.

[244] Patiño C, Kirchner T (2008). Estrés y coping en inmigrantes latinoamericanos residentes en Barcelona. Revista Iberoamericana de Psicología Ciencia y Tecnología.

[245] Qureshi A, Revollo HW, Martinena P, Collazos F, Ramos M, Dip E (2010). Stress and coping in the hospitalized Latin American immigrant patient. J Psychosom Res.

[246] Béné C, Godfrey-Wood R, Newsham A, Davies M (2012). Resilience: new utopia or New Tyranny? Reflection about the potentials and limits of the concept of resilience in relation to vulnerability reduction programmes.

[247] Lindert J, Schinina G, Bernd R, Mladovsky P, Devillé W, Rijks B, Petrova-Benedict R, McKee M (2011). Mental health of refugees and asylum-seekers. Migration and health in the European Union.

[248] Crijnen AA, Bengi-Arslan L, Verhulst FC (2000). Teacher-reported problem behaviour in Turkish immigrant and Dutch children: a cross-cultural comparison. Acta Psychiatr Scand.

[249] Wingate MS, Alexander GR (2006). The healthy migrant theory: variations in pregnancy outcomes among US-born migrants. Soc Sci Med.

[250] Requena-Mendez A, Albajar-Vinas P, Angheben A, Chiodini P, Gascon J, Munoz J (2014). Health policies to control chagas disease transmission in European countries. PLoS Negl Trop Dis.

[251] Zimmerman C, Kiss L, Hossain M (2011). Migration and health: a framework for 21st century policy-making. PLoS Med.

[252] Ingleby D (2012). Migration, ethnicity and the ‘social determinants of health’ agenda. Psychosocial Intervention/Intervención Psicosocial.

[253] Borrell C, Pons-Vigues M, Morrison J, Diez E (2013). Factors and processes influencing health inequalities in urban areas. J Epidemiol Community Health.

