# Supplementary material for: Hispano-Americans in Europe: what do we know about their health status and determinants? A scoping review
Source: BMC Public Health. 2015 May 7;15:472. doi: 10.1186/s12889-015-1799-x (PMC4430018; doi:10.1186/s12889-015-1799-x)
Supplement: Additional file 2: — Re-migration flows of Hispano-Americans initially based in Spain (2008–2013). [file 12889_2015_1799_MOESM2_ESM.doc]

**Additional file 2. Re-migration flows of Hispano-Americans initially based in Spain (2008-2013)**

**
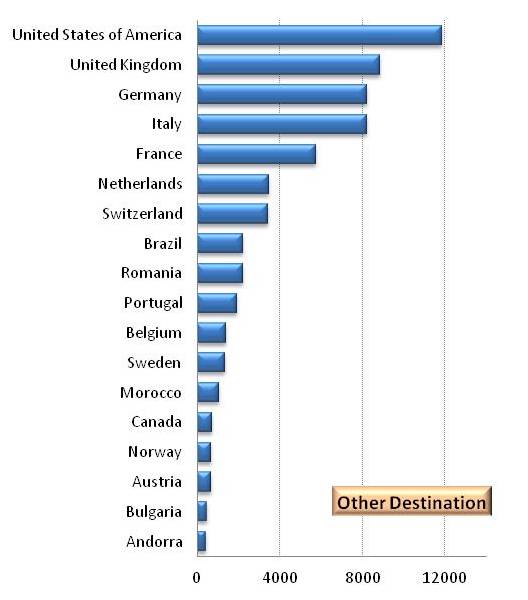
**

Source: Domingo A, based on data from the Spanish Statistics Institute
