# Supplementary material for: Hispano-Americans in Europe: what do we know about their health status and determinants? A scoping review
Source: BMC Public Health. 2015 May 7;15:472. doi: 10.1186/s12889-015-1799-x (PMC4430018; doi:10.1186/s12889-015-1799-x)
Supplement: Additional file 3: — Electronic search conducted via OVID on the 22/05/2014. [file 12889_2015_1799_MOESM3_ESM.doc]

**Additional file 3.** Electronic search conducted via OVID on the 22/05/2014

DATA BASES: Embase 1980 to 2014 Week 20, Global Health 1910 to 2014 Week 19**,** Ovid MEDLINE(R) 1946 to May Week 2 2014, Social Policy and Practice 201404

Citations yielded: 4,216.

1. "inmigr*".sh,ti.

2. "*immigr*".sh,ti.

3. "emigr*".sh,ti.

4. "migrant*".sh,ti.

5. remove duplicates from 1

6. 2 or 3 or 4 or 5

7. "central america*".ab,sh,ti.

8. "south america*".ab,sh,ti.

9. "southamerica*".ab,sh,ti.

10. "latin america*".ab,sh,ti.

11. "latinamerica*".ab,sh,ti.

12. "ibero america*".ab,sh,ti.

13. "iberoamerica*".ab,sh,ti.

14. "iberian america*".ab,sh,ti.

15. "hispano america*".ab,sh,ti.

16. "hispanoamerica*".ab,sh,ti.

17. "hispanic america*".ab,sh,ti.

18. "andean*".ab,sh,ti.

19. remove duplicates from 9

20. remove duplicates from 11

21. remove duplicates from 12

22. remove duplicates from 13

23. remove duplicates from 16

24. remove duplicates from 18

25. 7 or 8 or 10 or 14 or 15 or 17 or 19 or 20 or 21 or 22 or 23 or 24

26. "argentin*".ab,sh,ti.

27. "bolivia*".ab,sh,ti.

28. "chile*".ab,sh,ti.

29. "colombia*".ab,sh,ti.

30. "costa ric*".ab,sh,ti.

31. "costaric*".ab,sh,ti.

32. remove duplicates from 31

33. "cuba*".ab,sh,ti.

34. "ecuador*".ab,sh,ti.

35. "equador*".ab,sh,ti.

36. "salvador*".ab,sh,ti.

37. "guatemal*".ab,sh,ti.

38. "hondur*".ab,sh,ti.

39. remove duplicates from 38

40. "mexic*".ab,sh,ti.

41. "nicarag*".ab,sh,ti.

42. remove duplicates from 41

43. "panam*".ab,sh,ti.

44. "paraguay*".ab,sh,ti.

45. remove duplicates from 44

46. "peru*".ab,sh,ti.

47. "puerto ric*".ab,sh,ti.

48. "puertoric*".ab,sh,ti.

49. "dominican*".ab,sh,ti.

50. remove duplicates from 49

51. "uruguay*".ab,sh,ti.

52. "venezuel*".ab,sh,ti.

53. 26 or 27 or 28 or 29 or 30 or 32 or 33 or 34 or 35 or 36 or 37 or 39 or 40 or 42 or 43 or 45 or 46 or 47 or 48 or 50 or 51 or 52

54. 25 or 53

55. 6 and 54

56. limit 55 to yr="1985 -Current"

57. limit 56 to abstracts

58. limit 57 to yr="1990 -Current"

59. remove duplicates from 58
