# Supplementary material for: Hispano-Americans in Europe: what do we know about their health status and determinants? A scoping review
Source: BMC Public Health. 2015 May 7;15:472. doi: 10.1186/s12889-015-1799-x (PMC4430018; doi:10.1186/s12889-015-1799-x)
Supplement: Additional file 4: — Studies on HIV. [file 12889_2015_1799_MOESM4_ESM.doc]

**Additional file 4. Studies on HIV**

| Study reference | Location | Participants  ***N;CO*** | Study design | Trans-  national | Condition | Key findings |
| --- | --- | --- | --- | --- | --- | --- |
| 1.Belza MJ et al.,2004 | SPAIN | *N=555;vc* | Quantitative-CS | NO | HIV, other STIs | HIV prevalence in HA female sex workers: 0.2%  Frequency of other STIs: moderate  High reported frequency of condom use and breakage |
| 2.Belza MJ et al.,2005 | SPAIN | *N=243;vc* | Quantitative-CS | NO | HIV | HIV prevalence in HA male sex workers > local male sex workers (16% *vs* 9%) |
| 3.Bermudez MP et al.,2011 | SPAIN | *N=984;vc* | Quantitative-CS | NO | HIV | Risk HIV infection in HAs > locals. Adolescent HAs had more HIV misconceptions and negative attitudes about condoms than locals |
| 4.Caro-Murillo AM et al.,2009 | SPAIN | *N=400;vc* | Quantitative-  Cohort | NO | HIV | HIV+ hospital clients who are foreign-born: 1/3 (mostly HA). Transmission in HAs: homo/bisexual (54.5%), hetero men (18%), hetero female (22%) |
| 5.Del Amo J et al.,2010 | EUROPE | *N=456;vc* | Quantitative-CS | NO | HIV | Total AIDS reports in migrants: 35%. AIDS cases increased in people from Latin-America by 50% between 1999-2006. Increasing % of Latin-Americans amongst AIDS reports in MSM (3% in 1999 *vs* 7.5% in 2006). Most common origin of AIDS cases: SSA, except for Spain, where Latin-Americans outnumber SSAs |
| 6.Dougan S et al.,2005 | BRITAIN | *N=46;vc* | Quantitative-CS | NO | HIV | South/Central Americans in new HIV diagnosis amongst foreign born: 11% (mostly white or mixed ethnic origin). Likely infected in the UK: 39% |
| 7.Elford J et al.,2012 | BRITAIN | *N=136;vc* | Quantitative-CS | NO | HIV | Self-reported HIV sero-positivity higher in South/Central American MSM as compared to British men (19% *vs* 13%) and other migrants. Reported HIV test uptake in South/Central American MSM (90% ) > white English (65%) and other migrant groups. No risk behaviour differences in MSM of different ethnicities |
| 8.Folch C et al.,2009 | SPAIN | *N=98;vc* | Quantitative-CS | NO | HIV, other STIs | Immigrant MSM, especially HAs, more likely unprotected anal sex *vs* local MSM |
| 9.Giuliani M et al.,2004 | ITALY | *N=927;vc* | Quantitative-CS | NO | HIV, other STIs | HIV-1 prevalence in South-Americans diagnosed with STI > locals (17% *vs* 9%) |
| 10.Guillen MS et al.,2005 | SPAIN | *N=17;vc* | Quantitative-CS | NO | HIV | 1/3 of HIV children diagnosed with HIV are foreign born, a half HA |
| 11.Guillen S et al.,2012 | SPAIN | *N=14;vc* | Quantitative-CS | NO | HIV | HIV diagnosis in HA children decreased between 1997-2009 |
| 12.Gutierrez M et al.,2004 | SPAIN | *N=139;mostly Ecuador* | Quantitative-CS | NO | HIV, other STIs | HIV rates in sex workers differed markedly by sex and reached 23% in transsexual females. High rates of syphilis in Ecuadorian sex workers (16%), mostly transsexual |
| 13.Holguín A et al.,2008 | SPAIN | *N=232;vc* | Quantitative-CS | NO | HIV | Total HIV infections diagnosed in foreign born: 50%(2007)  % of total HIV infections diagnosed in HAs increasing: 17%(2000) - 22%(2007)  Non-B subtypes increasing, particularly in HAs: 11%(2000) - 20%(2007) |
| 14.Hoyos J et al.,2013 | SPAIN | *N=1,422;n/a* | Quantitative-CS | NO | HIV | No difference in risks behaviours in HAs *vs* Spanish. Spanish testing at mobile testing units more likely to be first testers than HAs. Main reason for not testing: low risk perception. Fear of discrimination not relevant barrier |
| 15.Llenas-García J et al.,2012 | SPAIN | *N=145;vc* | Quantitative-CS | NO | HIV, Chagas | Prevalence of Chagas in HIV+ Bolivians: 16.7%. Units caring for HIV patients from areas were Chagas is prevalent should implement screening protocols |
| 16.Monge S et al.,2013 | SPAIN | *N=1,221;vc* | Quantitative-  Cohort | NO | HIV | Most HIV+ HAs are MSM. Delayed diagnosis in HAs > locally born. No differences in immunological and viral responses |
| 17.Palacio V et al.,2002 | SPAIN | *N=2,054;vc* | Quantitative-CS | NO | HIV | % total HIV infections diagnosed in foreign born: 35%(2000)  Higher HIV prevalence in both MSM (18%) and heterosexual HA men (4.5%) *vs* locals. HIV prevalence in HAs men > women (11.3% *vs* 0.3%) |
| 18.Pérez-Molina JA et al.,2009 | SPAIN | *N=469;vc* | Quantitative-CS | NO | HIV | HIV prevalence in HAs < SSA (3% *vs* 6%) but delayed diagnosis higher in HAs (80%) *vs* SSAs (40%). ¼ HAs did not start treatment when indicated |
| 19.Ríos E et al.,2009 | SPAIN | *N=129;vc* | Quantitative-CS | NO | HIV, Other STIs | ½ HAs believed there was a preventive HIV vaccination and held misconceptions about transmission. 53% had tested for HIV, with frequent repeated testing |
| 20.Rodríguez-Guardado A et al.,2011 | SPAIN | *N=14;vc* | Quantitative-CS | NO | HIV, Chagas | Reactivation of Chagas disease in HIV+ HA patients may occur. Serological test for Chagas disease to be considered in this population |
| 21.Romero A et al.,2012 | SPAIN | *N=210;vc* | Quantitative-CS | NO | HIV | Highest percentage of recent infection in HAs (28% *vs* 23% in locals and 12% in SSAs). MSM amongst new HIV cases in HAs: 68% |
| 22.Romero A et al.,2009 | SPAIN | *N=62;vc* | Quantitative-CS | NO | HIV, other STIs | Most infections amongst migrants appear to be locally acquired |
| 23.Spizzichino L et al.,1998 | ITALY | *N=79;vc* | Quantitative-CS | NO | HIV | HIV prevalence in Colombian transsexuals: 20%. Counselling strategies useful to promote safe sex amongst HA transsexual sex workers at drug treatment centre |
| 24.Spizzichino L et al.,2001 | ITALY | *N=226;vc* | Quantitative-CS | NO | HIV | High HIV prevalence in Colombian transsexuals: 22%. Increased reported regular use of condoms after counselling (from 72% to 87%) |
| 25.Suligoi B et al.,1997 | ITALY | *N=537;vc* | Quantitative-CS | NO | HIV, other STIs | Very high HIV prevalence amongst Central/South-American MSM attending STI clinics: 78% in injecting drug users (IDUs), 40% in non-IDUs |
| 26.Thierfelder C et al.,2012 | SWITZERLAND | *N=306;vc* | Quantitative-  Cohort | NO | HIV,other STIs | Non participation in cohort study more likely amongst migrants including Latin-American women. Latin-American more likely to be lost to follow-up.  Syphilis disproportionally high in Latin-Americans: 20% *vs* 9% average |
| 27.Van Haastrecht HJA et al.,1993 | The netherlands | *N=63;mainly Colombia and Dominican* | Quantitative-CS | NO | HIV, other STIs | Frequent inconsistent condom use in HA sex workers |
| 28.Yebra G et al.,2011 | SPAIN | *N=114;vc* | Quantitative-CS | NO | HIV | Transmitted drug resistant HIV tends to decrease in HAs |
| 29.Zaccarrelli M et al.,2004 | ITALY | *N=289;*  *Colombia* | Quantitative-CS | NO | HIV | Stable HIV prevalence in Colombian transsexuals: 17% (1992-3) *vs* 16% (2002-2003). Increased reported regular condom use: 93% in 2000-2001 |

*Acronyms used: CO (country of origin); vc (various countries); CS (cross-sectional); HIV (human immunodeficiency virus); HA (Hispano American); STIs (sexually transmitted infections); HAs (Hispano Americans); MSM (men who have sex with other men); SSA (Sub-Saharan Africa); n/a (not available)*
