# Supplementary material for: Hispano-Americans in Europe: what do we know about their health status and determinants? A scoping review
Source: BMC Public Health. 2015 May 7;15:472. doi: 10.1186/s12889-015-1799-x (PMC4430018; doi:10.1186/s12889-015-1799-x)
Supplement: Additional file 5: — Studies on other STIs. [file 12889_2015_1799_MOESM5_ESM.doc]

**Additional file 5. Studies on other STIs**

| Study reference | Location | *Participants*  ***N;CO*** | Study design | Trans-  national | Condition | Key findings |
| --- | --- | --- | --- | --- | --- | --- |
| 1.del Amo et al.,2005 | SPAIN | *N=650;vc* | Quantitative-CS | NO | HPV  various STIs | HPV prevalence in migrant female SW: 39% (including HAs)  STI prevalence: 12% (Colombians/Ecuadorians), 14% (Caribbeans) |
| 2.González C et al.,2006 | SPAIN | *N=122;vc* | Quantitative-CS | NO | HPV | HPV infection in HAs (21%-27%) *>* Spanish (8%) |
| 3.Tornesello ML et al.,2011 | ITALY | *N=8;vc* | Quantitative-CS | NO | HPV | HPV prevalence in Latin-Americans: 62% |
| 4.Ramos JM et al.,2011 | SPAIN | *N=520;vc* | Quantitative-CS | NO | HTLV | HTLV prevalence in pregnant HAs (0.6%) > average in pregnant migrant women (0.2%) |
| 5.Treviño A et al.,2009 | SPAIN | *N=797;vc* | Quantitative-CS | NO | HTLV | HTLV prevalence in pregnant HAs > local women (<0.02%) |
| 6.Treviño A et al.,2011 | SPAIN | *N=1,579;vc* | Quantitative-CS | NO | HTLV | HTLV prevalence in pregnant HAs (0.3%) > average pregnant migrant women (0.2%) |
| 7.Treviño A et al.,2012 | SPAIN | *N=316;vc* | Quantitative-CS | NO | HTLV | HTLV prevalence stabilised in Spain but increased HTLV1 associated to HA migration |
| 8.González-López J et al.,2009 | SPAIN | *N=101;vc* | Quantitative-LN | NO | Syphilis | Syphilis incidence rose 223% (2003-2007) affecting MSM, HIV+, HAs |
| 9.De Sanjosé S et al.,2002 | SPAIN | *N=~76;vc* | Quantitative-CS | NO | KS | KS prevalence in SW, mostly HAs (16%) > general pop (8%) |
| 10.Wolff H et al.,2008 | SWITZERLAND | *N=137;vc* | Quantitative-Cohort | NO | CT | CT prevalence in undocumented migrants mostly originating from Latin-America (13%) > documented migrants (4%). No contraception use in undocumented Latin-Americans (23%) > documented (5%) |
| 11.Zehender G et al.,2004 | ITALY | *N=109;mainly Peru* | Quantitative-CS | NO | HTLV | HTLV prevalence in HAs > other migrant groups (26% in HIV+) Transsexual SWs particularly affected |

*Acronyms used: STIs (sexually transmitted infections); CO (country of origin); vc (various countries); CS (cross-sectional); HPV (human papilloma virus); SW (sex worker); HAs (Hispano Americans); HTLV (human T-lymphotropic virus); LN (longitudinal); MSM (men who have sex with other men); KS (karposi sarcoma); CT(chlamydia trachomatis)*
