# Supplementary material for: Hispano-Americans in Europe: what do we know about their health status and determinants? A scoping review
Source: BMC Public Health. 2015 May 7;15:472. doi: 10.1186/s12889-015-1799-x (PMC4430018; doi:10.1186/s12889-015-1799-x)
Supplement: Additional file 6: — Studies on Tuberculosis. [file 12889_2015_1799_MOESM6_ESM.doc]

**Additional file 6. Studies on Tuberculosis**

| Study reference | Location | Participants  ***N;CO*** | Study design | Trans-  national | Outcome measure | Key findings |
| --- | --- | --- | --- | --- | --- | --- |
| 1.Arce Arnaez A et al.,2005 | SPAIN | N=151;vc | Quantitative-CS | NO | TB cases | >50% TB cases identified in foreign-born are HA. Of the foreign-born, 74% developed tuberculosis within 5 years of arrival (51% within 2 years) |
| 2.Barbero BS et al.,2010 | SPAIN | N=75;vc | Quantitative-CS | NO | TB knowledge, attitudes and practices | High percentage of HAs with TB identified erroneous transmission routes, such as blood (42%), sexual fluids (50%) or eating utensils (81%) |
| 3.Borell S et al.,2010 | SPAIN | N=126;vc | Quantitative-CS | NO | TB cases | 58% TB cases identified in foreign-born are from HA. 50% of foreign-born TB patients developed the disease during first 2 years after arrival, mostly due to reactivation. Significant bidirectional transmission local-foreign born |
| 4.Giachino R et al.,2003 | ITALY | N=100;vc | Quantitative-CS | NO | TB cases | 44% migrant children with TB are HA |
| 5.Marco A et al.,2012 | SPAIN | N=37;vc | Quantitative-CS | NO | TB prevalence | ~50% HA inmates affected by latent TB. HAs more likely to be affected than locals |
| 6.Martin V et al.,2011 | SPAIN | N=142;vc | Quantitative-CS | NO | TB Incidence as AIDS defining | HAs more likely to develop TB as AIDS defining disease than locals |
| 7.Martinez-R A et al.,2006 | SPAIN | N=167;vc | Quantitative-CS | NO | TB cases | 43% TB cases identified in migrant children originate from HA |
| 8.Perone SA et al.,2005 | SWITZERLAND | N=15;vc | Quantitative-CS | NO | TB cases | 68% TB cases identified in undocumented foreign-born populations originate from HA, mostly young women in domestic services |
| 9.Ramos JM et al.,2002 | SPAIN | N=148;vc | Quantitative-CS | NO | TB prevalence | TB prevalence (6%) < Sub-Saharans (19%), East Europeans (10%) |
| 10.Rapiti E et al.,1998 | ITALY | N=60;vc | Quantitative-CS | NO | TB prevalence | 5.4 times more likely to develop TB than other migrants |
| 11.Rodriguez NA et al.,2009 | SPAIN | N=351;vc | Quantitative-CS | NO | TB cases | >50% TB cases in foreign-born populations originate from HA. Transmission permeability among nationalities, including from autochthonous to foreigners |
| 12.Ruiz López FJ et al.,2006 | SPAIN | N~66;mostly Ecuadorians | Quantitative-CS | NO | TB prevalence | 42% TB cases in immigrants (mostly Ecuadorians) with < 1 year in Spain |
| 13.Wolff H et al.,2010 | Switzerland | N=725;vc | Quantitative-CS | NO | TB-related fibrotic signs | Latin-Americans with regular status 2.7 times more likely to have TB signs than regular migrants from other areas. Latin-Americans with irregular status 5.5 times more likely to have TB signs *vs* irregular migrants from other areas |
| 14.Yuguero O et al.,2012 | SPAIN | N=17;vc | Quantitative-CS | NO | TB incidence and treatment compliance | Treatment compliance > locals and migrants’ average (82% *vs* 76% and 61%). TB incidence higher in migrants *vs* locals |

Acronyms used: TB (tuberculosis)*; CO (country of origin);* vc (various countries)*; CS (cross-sectional); HA (Hispano American); HAs (Hispano Americans)*
