# Supplementary material for: Hispano-Americans in Europe: what do we know about their health status and determinants? A scoping review
Source: BMC Public Health. 2015 May 7;15:472. doi: 10.1186/s12889-015-1799-x (PMC4430018; doi:10.1186/s12889-015-1799-x)
Supplement: Additional file 7: — Studies on other communicable diseases. [file 12889_2015_1799_MOESM7_ESM.doc]

**Additional file 7. Studies on other communicable diseases**

| Study reference | Location | Participants  ***N;CO*** | Study design | Trans-  national | Condition/topic addressed | Key findings |
| --- | --- | --- | --- | --- | --- | --- |
| 1.Baas MC et al.,2006 | THE NETHERLANDS | *N=18;n/a* | Quantitative-CS | NO | Imported malaria | Cases of vivax malaria acquired in Latin-America: 32% |
| 2.Bruguera M et al.,2006 | SPAIN | *n/app* | Literature review | NO | Hepatitis C | Prevalence of Hepatitis C in HAs < locals |
| 3.Capretti MG et al.,2014 | ITALY | *N=138,n/a* | Quantitative-CS | NO | Toxoplasma gondii | Toxoplasma gondii in HA women > locally born  Prenatal screening effective |
| 4.Gergely AE et al.,2011 | FRANCE | *N=72;n/a* | Quantitative-CS | NO | Hepatitis A | Latin-Americans with Hepatitis A antibodies: 57% |
| 5.Huerga Aramburu et al.,2004 | SPAIN | *N=34;n/a* | Quantitative-CS | NO | Infectious diseases in children | Frequent co-infection with various infectious diseases even in asymptomatic children |
| 6.López-Vélez et al.,2003 | SPAIN | *N=163;n/a* | Quantitative-CS | NO | Various infectious diseases | Frequent concomitant diagnoses: 3 or more in 14% HAs. Latent and active TB in HAs (47% and 10%) > SSAs (44% and 4%) |
| 7.Masvidal RM et al.,2010 | SPAIN | *N=304;vc* | Quantitative-CS | NO | Hepatitis A and C | Low prevalence of HCV antibodies. Systematic screening not recommended. HAV antibodies in 35% Latin-American children |
| 8.Muñoz E et al.,2003 | SPAIN | *N=612;vc* | Quantitative-CS | NO | Vaccine coverage | Against common belief, vaccine coverage is high |
| 9.Navarro GC et al.,2011 | SPAIN | *n/app* | Literature review | NO | Vaccination needs | All first year resident physicians from Peru who do not have vaccination documents should be vaccinated as if they were a non vaccinated adult |
| 10.Ramos JM et al.,2011 | SPAIN | *N=535;vc* | Quantitative-CS | NO | Toxoplasma gondii | Seroprevalence of Toxoplasma gondii in HA women (43%) > locals (12%) |
| 11.Ramos JM et al.,2012 | SPAIN | *N=564;vc* | Quantitative-CS | NO | Rubella | Low immunity against Rubella in HA migrants but marked differences by country of origin. Argentineans had immunity rates similar to locals |
| 12.Roca C et al.,2003 | SPAIN | *N=1,714;vc* | Quantitative-CS | NO | Neurocysticercosis | Most neurocysticercosis cases originated in HA (74%). Study of seizures/neurological symptoms needed in all patients with attention to HAs |
| 13.Santiago B et al.,2012 | SPAIN | *N=100;vc* | Quantitative-CS | NO | Infectious diseases | T.Cruzi in 12% Bolivian pregnant women. Highest T.pallidum rates in HAs |
| 14.Sanz JC et al.,2004 | SPAIN | *N=15;vc* | Quantitative-CS | NO | Rubella | Rubella vaccination introduced in Hispano American in the 90s so HA migrant may not be immunised. Due prevention strategies needed |
| 15.Terraza S et al.,2001 | SPAIN | *N=5;vc* | Quantitative-CS | NO | Neurocysticercosis | Neurocysticercosis cases mostly acquired in HA |
| 16.Turrientes MC et al.,2011 | SPAIN | *N=634;vc* | Quantitative-CS | NO | Visceral larva | Eosinophilia in 21% patients. Frequent polyparasitism. Visceral larva should be included in the differential diagnosis of eosinophilia in migrants from tropical areas with respiratory/abdominal symptoms |
| 17.Valerio L et al.,2009 | SPAIN | *N=5,773;n/a* | Quantitative-CS | NO | Varicella | Originating from HA may account for vulnerability to varicella. Vaccination of immigrants at high risk could be recommendable |
| 18.Valerio LL et al.,2008 | SPAIN | *N=329,n/a* | Quantitative-CS | NO | Hepatitis B | Prevalence in HAs < locals. Migrants’ screening recommended except HAs |
| 19.Villari P et al.,2001 | ITALY | *N=87; Peru,*  *Dominic. Rep* | Quantitative-CS | NO | Hepatitis G | High Hepatitis Prevalence in HAs (19.5%), very high in Dominicans (26.5%). Widespread in general population (12.6%). Some cases locally acquired |
| 20.Zammarchi L et al.,2013 | VC | *N~66,mostly Ecuadorians* | Systematic literature review | NO | Cysticercosis, Taenia solium Taeniasis | Most imported cases acquired in HAs and diagnosed in Spain, France, Italy and the UK. Highest mean hospitalization rates in Ecuadorians, Bolivians and Peruvians. Diagnostic delay can be huge. Many neurosurgical operations due to low awareness and lack of experience managing the disease in Europe, probably avoidable. Transfer of knowledge from experts in endemic areas needed. Insufficient diagnostic and treatment options |

Acronyms used: *CO (country of origin);* n/a (not available); *CS (cross-sectional); n/app (not applicable); HAs (Hispano Americans)*; *HA (Hispano American)*; *SSA (Sub-Saharan Africa);* vc (various countries)
