# Supplementary material for: Hispano-Americans in Europe: what do we know about their health status and determinants? A scoping review
Source: BMC Public Health. 2015 May 7;15:472. doi: 10.1186/s12889-015-1799-x (PMC4430018; doi:10.1186/s12889-015-1799-x)
Supplement: Additional file 8: — Studies on cardio-vascular diseases and risk factors. [file 12889_2015_1799_MOESM8_ESM.doc]

**Additional file 8. Studies on cardio-vascular diseases and risk factors**

| Study reference | Location | *Participants*  ***N;CO*** | Study design | Trans-  national | Condition | Key findings |
| --- | --- | --- | --- | --- | --- | --- |
| 1.Franch-Nadal J et al.,2013 | SPAIN | *N=172;n/a* | Quantitative-CS | NO | DM, Obesity | Migrants with DM younger than locals and with fewer complications. Obesity in HAs with DM > locals and other migrants |
| 2.González-López JR et al.,2013 | SPAIN | *N=33;vc* | Quantitative-CS | NO | CVD morbidity/RF | CVD morbidity and risk factors>previously reported: 9% DM, 15% high cholesterol, 23.5% HT, 9% coronary accidents |
| 3.Jianguang J et al.,2010 | SWEDEN | *N=11;vc* | Quantitative-CS | NO | DM | DM risk in HA adoptees and “second generation” migrants < locals |
| 4.Johansson-Kark M et al.,2002 | SWEDEN | *N=614;vc* | Quantitative-CS | NO | OW | OW in adopted HA children (21.5%) > locals (14%) and other adoptees  HAs, particularly Chileans, may be genetically susceptible to OW |
| 5.Khan FA et al.,2004 | SWEDEN | *N=338;Chile* | Quantitative-LN | NO | Stroke incidence | Stroke risk in Chileans~locals. Marked differences across migrant groups |
| 6.Lozano Sánchez ML et al.,2013 | SPAIN | *N=298;vc* | Quantitative-CS | NO | CVD risk | Dramatic increase in CVD risk with length of migration  CVD risk in HAs residing > 8 years in Spain (49%) > locals (28%) |
| 7.Marín-Guerrero AC et al.,2010 | SPAIN | *N=589;vc* | Quantitative-CS | NO | Obesity | Obesity in HAs (13%) > locals (9.5%) and particularly high in men (17%) |
| 8.Regidor E et al.,2008 | SPAIN | *N=79;vc* | Quantitative-CS | NO | CVD mortality | Low CVD mortality in South American men  High mortality from cerebrovascular disease in Central Americans and Caribbeans |
| 9.Regidor E et al.,2009a | SPAIN | *N=810;vc* | Quantitative-CS | NO | CVD mortality | Risk of mortality from ischemic heart disease in South Americans < locals  Risk of cerebrovascular mortality in women form the Caribbean > locals |
| 10.Regidor E et al.,2009b | SPAIN | *N=230;vc* | Quantitative-CS | NO | CVD mortality | CVD mortality in South American < locals/other migrants  Cerebrovascular mortality in Central Americans/Caribbeans > locals/other migrants |
| 11.Roca Vilalta M et al.,2006 | SPAIN | *N=10;vc* | Quantitative-CS | NO | DM | DM prevalence in HAs below 60 years < locals/other migrants |
| 12.Serrano-Ríos M et al.,1999 | VC | *n/ap* | Literature review | YES | DM | DM is caused by both genetic and environmental factors |
| 13.Söderström U et al.,2012 | SWEDEN | *N=28,498;vc* | Quantitative-CS | NO | DM | “Second generation” migrants with parents from low DM prevalence areas have higher risk than adoptees from same areas. Low DM risk persists in adoptees from low prevalence areas. Exposures in utero/early infancy are RF for DM |
| 14.Wändeil PE et al.,2004 | SWEDEN | *N=564;Chile* | Quantitative-CS | NO | BMI | BMI higher in Chileans *vs* locals after adjusting for age, education and life-styles |
| 15.Zöller B et al.,2012 | SWEDEN | *N=166;*  *mostly Chile* | Quantitative-LN | NO | Incidence of VT and PE | Risk for VT and PE in 1^st^-generation HAs < Swedish. Decreased risk maintained in “2^nd^ generation” for VT but not PE. Both acquired and inherited factors contribute to VT/PE |

*Acronyms used: CO (country of origin); n/a (not available); CS (cross-sectional); DM (diabetes mellitus); HAs (Hispano-Americans); vc (various countries); CVD (cardiovascular disease); RF (risk factors); HT (hypertension); HA (Hispano-American); OW (overweight); LN (longitudinal); BMI (body mass index); VT (venous thrombosis); PE (pulmonary embolism)*
