# Supplementary material for: Hispano-Americans in Europe: what do we know about their health status and determinants? A scoping review
Source: BMC Public Health. 2015 May 7;15:472. doi: 10.1186/s12889-015-1799-x (PMC4430018; doi:10.1186/s12889-015-1799-x)
Supplement: Additional file 9: — Studies on cancer. [file 12889_2015_1799_MOESM9_ESM.doc]

**Additional file 9. Studies on cancer**

| Study reference | Location | *Participants*  ***N;CO*** | Study design | Trans-  national | Condition | Key findings |
| --- | --- | --- | --- | --- | --- | --- |
| 1.Azerkan F et al.,2008 | Sweden | *N=36,795;vc* | Quantitative-Cohort | NO | CC | Higher relative risk of CC in migrants from all countries *vs* Swedish, particularly high in Central Americans (RR: 2.5%) |
| 2.Beiki O et al.,2009 | SWEDEN | *N=33,650;vc* | Quantitative-Cohort | NO | CC | HA women aged > 50 at increased risk of CC |
| 3.González RY et al.,2003 | SPAIN | *N=153;vc* | Quantitative-CS | NO | CC | CC screening in HAs < locals (25% *vs* 44%) |
| 4.Hemminki K et al.,2002 | SWEDEN | *N=6,517;Chile* | Quantitative-CS | NO | Various types of cancer | Chilean men risk for testicular cancer > locals  Nervous system tumours < locals |
| 5.Hemminki K et al.,2010 | SWEDEN | *N=48;vc, mainly Chile* | Quantitative-CS | NO | Liver and gallbladder cancer | Chileans at increased risk of gallbladder cancer |
| 6.Hemminki K et al.,2011 | SWEDEN | *N=244;vc* | Quantitative-CS | NO | Breast cancer | HAs risk of breast cancer < locals |
| 7.Mousavi SM et al.,2011a | SWEDEN | *N=52;vc* | Quantitative-CS | NO | Thyroid cancer | HAs females risk of papillary thyroid cancer > locals |
| 8.Mousavi SM et al.,2011b | SWEDEN | *N=36;vc, mainly Chile* | Quantitative-CS | NO | Nervous system cancer | Chileans’ risk of brain glioma and meningioma < locals |
| 9.Mousavi SM et al.,2012 | SWEDEN | *N=31,525;vc, mainly Chile* | Quantitative-Cohort | NO | Cervical, endometrial and ovarian cancer | Ovarian cancer in Chileans < locals, maintained in “second generation” |
| 10.Mousavi SM et al.,2013 | SWEDEN | *N=4,964;vc, mainly Chile* | Quantitative-CS | YES | Various types of cancer | Chilean migrants’ risk for all cancer types < residents at country of origin. Chileans risk for stomach and testicular cancer > locals  Chileans risk for colon, nervous system cancer and non-Hodgkin’s lymphoma < locals |
| 11.Nacif-Gomera ML et al.,2013 | SPAIN | *N=55;vc* | Quantitative-CS | NO | Various types of cancer | Support of psychologists and social workers improves psychosocial variables in migrant children with cancer but challenges remain |
| 12.Puigpinos R et al.,2012 | SPAIN | *N=257;vc* | Quantitative-CS | NO | Mammography uptake | Mammography uptake in HA women > most migrant groups but < locals |
| 13.Sanz-Barbero B et al.,2011 | SPAIN | *N=995;vc* | Quantitative-CS | NO | Mammography, cervical smear uptake | HAs less likely to undergo mammogram than locals but equally likely to undergo cervical smears |

*Acronyms used: CO (country of origin); vc (various countries); CC (cervical cancer); HA (Hispano American); CS (cross-sectional); HAs (Hispano Americans)*
