# Supplementary material for: Hispano-Americans in Europe: what do we know about their health status and determinants? A scoping review
Source: BMC Public Health. 2015 May 7;15:472. doi: 10.1186/s12889-015-1799-x (PMC4430018; doi:10.1186/s12889-015-1799-x)
Supplement: Additional file 10: — Studies on other non-communicable conditions. [file 12889_2015_1799_MOESM10_ESM.doc]

**Additional file 10. Studies on other non-communicable conditions**

| Study reference | Location | Participants  ***N;CO*** | Study design | Trans-  national | Condition(s) addressed | Key findings |
| --- | --- | --- | --- | --- | --- | --- |
| 1.Domínguez-Ortega J et al.,2011 | SPAIN | *N=41;n/a* | Quantitative-CS | NO | Allergies | Important difference in prevalence of allergies in migrants *vs* locals. Particularly high rates in HAs. Most relevant allergen: grass pollen |
| 2.Tedeschi A et al.,2003 | ITALY | *N=77;vc* | Quantitative-CS | NO | Allergies | Increased risk of allergies/asthma in HAs  Symptoms appearing mostly after migration  High variability by country of origin and sex |
| 3.Burastero SE et al.,2011 | ITALY | *N=250;n/a* | Quantitative-CS | NO | Allergies | HAs disproportionally affected by rhinitis/asthma |
| 4.Esteban-Vasallo MD et al.,2009 | SPAIN | *N=206,288;*  *n/a* | Quantitative-CS | NO | Chronic diseases | Chronic diseases less frequent in migrant populations *vs* locals but back-pain and migraine more frequent in HA males. Anaemia, constipation and migraine more common in HA females *vs* locals |

Acronyms used: *CO (country of origin);* n/a (not available); *CS (cross-sectional); HAs (Hispano Americans)*; vc (various countries)*; HA (Hispano American)*
