# Supplementary material for: Hispano-Americans in Europe: what do we know about their health status and determinants? A scoping review
Source: BMC Public Health. 2015 May 7;15:472. doi: 10.1186/s12889-015-1799-x (PMC4430018; doi:10.1186/s12889-015-1799-x)
Supplement: Additional file 11: — Studies on emotional health. [file 12889_2015_1799_MOESM11_ESM.doc]

**Additional file 11. Studies on emotional health**

| Study reference | Location | Participants  ***N;CO*** | Study design | Outcome  measure | Trans-  national | Key findings |
| --- | --- | --- | --- | --- | --- | --- |
| 1.Alvarez del Arco D et al.,2013 | Spain | *N=534;Ecuador* | Quantitative-CS | PPC | NO | Ecuadorian women particularly vulnerable to interpersonal violence and the mental health consequences derived from it |
| 2.Aragona M et al.,2008 | ITALY | *N=301;30% South/Central America* | Quantitative-CS | Bradford Somatic Inventory score | NO | Female sex is a significant predictor of somatisation |
| 3.Aragona M et al.,2012 | ITALY | *N=1,329;vc* | Quantitative-CS | Bradford Somatic Inventory score | NO | Somatisation in HAs (30%) > Europeans (23%), Africans (21%) and Asians (16%). Particularly high in Peruvians (33%) |
| 4.Bayard-Burfield L et al.,2001 | SWEDEN | *N=571;Chile* | Quantitative-CS | Self-reported psychiatric illness/ psychotropic drugs use | NO | Chileans four times more likely to report psychiatric illness than Swedes. Strong association between ethnicity and self-reported psychiatric illness. The higher prevalence of mental risk factors in migrants can’t be explained solely by socio-economic determinants |
| 5.Cruz I et al.,2010 | Spain | *N=6,641;vc* | Quantitative-CS | Antidepressant drugs dispensed | NO | Lower prescription of antidepressants in immigrants *vs* natives. Highest rates in HA females (6.6%) but still < autochthonous (11%) |
| 6.del Amo J et al.,2011 | Spain | *N=1,122;Ecuador* | Quantitative-CS | PPC | NO | PPC prevalence higher in Spanish and Ecuadorian women *vs* men of same nationalities. Ecuadorian women more likely to be identified as PPC than Spanish females but Ecuadorian men had not higher PPC prevalence than Spanish men. Having university studies doubles PPC risk in Ecuadorians, which could be due to downward social mobility |
| 7.González Castro JL et al.,2011 | Spain | *N=81;Ecuador* | Quantitative-CS | Self-reported health | NO | Perceived discrimination, low income and female sex enhance mental distress. Support from family members at CO helps to cope with stress |
| 8.Herrero J et al.,2011 | SPAIN | *N=350;vc* | Quantitative-CS | SWB | NO | Social integration in the community positively correlates with SWB |
| 9.Jarrín I et al.,2013 | Spain | *N=568;Ecuador* | Quantitative-CS | PPC | NO | Prevalence of PPC: 24%. Ecuadorians in areas with higher ethnic density more likely to be PPC vs those living in low ethnic density neighbourhoods. Much of the effect explained by poverty of the area |
| 10.Kirchner T et al.,2010 | Spain | *N=295;vc* | Quantitative-CS | Self-perceived stress and depression  symptoms | NO | Strong sense of religiosity reported by males and females and negatively related with stress. Religiosity buffers relationship between stress and depression in women, which could be explained by social support from the religious community. Religiosity declined with length of stay |
| 11.Lipsicas CB et al.,2012 | EUROPE | *N=58,622;Chile* | Quantitative-CS | Suicide attempt rates | NO | Suicide attempt rates higher in most immigrant groups *vs* locals. Chilean migrants had the highest suicide attempt rates in spite of low rates reported in Chile |
| 12.Llàcer A et al.,2009 | Spain | *N=570;Ecuador* | Quantitative-CS | PPC | NO | Prevalence of PPC: 28%. High rates of perceived discrimination (>60%). Strong association between perceived discrimination and mental health may be exacerbated by financial strains. Participation in community groups/associations independently associated with poor mental health |
| 13.Pascual JC et al.,2008 | Spain | *N=475;vc* | Quantitative,  Longitudinal description | Diagnosis of borderline personality | NO | Borderline personality disorder diagnosed less frequently in migrants *vs* locals in an emergency psychiatric service. South Americans diagnosed more often than Asians and Sub-Saharans |
| 14.Patiño C et al.,2010 | Spain | *N=210;vc* | Quantitative-CS | Symptoms of psychological distress | NO | High level of stress and psychopathology symptoms in a non clinical population. Increase of psychological distress over time. Main stressors: not having a job and financial difficulties. Psychopathology symptoms and use of coping strategies > autochthonous. Use of avoidance coping strategies increases with length of residence. Immigration is psychologically stressful and contributes to mental health problems |
| 15.Qureshi A et al.,2013 | Spain | *N=614;vc* | Quantitative-CS | Psychiatric morbidity | NO | Migrants in general have not higher rates of psychiatric morbidity than locals but Latin-Americans do (37% *vs* 31%) |
| 16.Revollo HW et al.,2011 | Spain | *N=414;vc* | Quantitative-CS | Depression and anxiety symptoms, acculturative stress, psychiatric pathology | NO | General psychosocial stress associated with psychopathology. Relatively low levels of perceived discrimination reported. Perceived discrimination and homesickness not associated with psychopathology. Prevalence of depression and anxiety not different from that in the general population |
| 17.Ruiz Hernández JA et al.,2011 | Spain | *N=692;vc* | Mixed methods  (Qual. and Quant.) | Perceived stress | NO | The acculturation process inherent to migration leads to high stress related to nostalgia, family break-up and perceived discrimination |
| 18.Sendra-Gutierrez JM et al.,2012 | Spain | *N=69;vc* | Quantitative,  Longitudinal description | Psychiatric consultations | NO | Psychiatric morbidity is associated with being a migrant. Incidence of new psychiatric consultations in HAs > locals |
| 19.Sundquist J et al.,2000 | SWEDEN | *N=571;Chile* | Mixed methods  (Qual. and Quant.) | Psychological distress,  Psychosomatic complaints | NO | Post migration stressors seem stronger risk factors for poor mental health than exposure to violence before migration. Headache complains in 50% Chilean women |
| 20.Sundquist J et al.,1995 | SWEDEN | *N=11;Chile* | Qualitative | Individuals’ perceptions of the exile experience | YES | Experience of exile increases vulnerability to psychological distress and physical disease |

Acronyms used: CO (country of origin); CS (cross-sectional); PPC (possible psychiatric case); vc (various countries); HAs (Hispano-Americans); HA (Hispano-American); SWB (subjective well-being)
