# Supplementary material for: Hispano-Americans in Europe: what do we know about their health status and determinants? A scoping review
Source: BMC Public Health. 2015 May 7;15:472. doi: 10.1186/s12889-015-1799-x (PMC4430018; doi:10.1186/s12889-015-1799-x)
Supplement: Additional file 12: — Studies on maternal and child health. [file 12889_2015_1799_MOESM12_ESM.doc]

**Additional file 12. Studies on maternal and child health**

| Study reference | Location | Participants  ***N;CO*** | Study design | Trans-  national | Outcome measure | Key findings |
| --- | --- | --- | --- | --- | --- | --- |
| 1.Agudelo-Suárez AA et al.,2009 | SPAIN | *N=92,743;vc* | Quantitative-CS | NO | PTB, LBW | PTB in SAs (7.3%) and CAs (7.8%) < locals (7.9%)  LBW in SAs (2%) and CAs (2.6%) < locals (3.2%) |
| 2.Barona-Vilar C et al.,2013 | SPAIN | *N=26;Bolivia/Ecuador* | Qualitative | NO | Perceptions of motherhood and maternal health care | Some women perceived that prenatal controls offer little benefit. Barriers to accesing services included irregular employment and inflexible apppointment time tables |
| 3.Bernis C et al.,2013 | SPAIN | *N=3,701;vc* | Quantitative-CS | NO | CS use | CS in HAs > locals |
| 4.Ekéus C et al.,2010 | SWEDEN | *N=1,899;Chile* | Quantitative-CS | NO | EDA use | EDA in Chileans (52%) > locals/other migrants |
| 5.Gredilla E et al.,2008 | SPAIN | *N=136;vc* | Quantitative-CS | NO | EDA use | High satisfaction with EDA in local and migrant women |
| 6.Hernández-Rivas E et al.,2012 | SPAIN | *N=71;vc* | Quantitative,  Prospective Cohort | NO | Various perinatal outcomes in women diagnosed with GDM | CS (53%) > locals (33.5%)  Higher frequency of adverse perinatal outcomes in HAs *vs* Caucasian women diagnosed with GDM |
| 7.Hernando V et al.,2005 | SPAIN | *N=82;vc* | Quantitative-CS | NO | Use of FP | High variability in reported use of FP methods, ranging from 100% in Colombians to 55% in Dominicans |
| 8.Jiménez-Puente A et al.,2012 | SPAIN | *N=2,426;vc* | Quantitative-CS | NO | EDA, CS use, BW | EDA (57%) < Spanish (61%)/EE (58%) > other migrants  CS (26%) > locals (21%); BW (3,350gr) > locals (3,231) |
| 9.Malin M et al.,2009 | FINLAND | *N=121;vc* | Quantitative-CS | NO | LBW,PTB,CS, Perinatal mortality | CS (31%) > locals (20%) and migrant average (20%)  PTB (6%) > locals (4.8%) and migrant average (5.2%)  LBW (4%) > locals (3%)  Newborn in intensive care (13%) > locals (9%)  Intubation of newborns (1.7%) > locals (0.9%)  Perinatal mortality (8‰) > locals (5‰) |
| 10.Merry L et al.,2013 | VC | *Not applicable* | Systematic review | NO | CS use | HA women have higher emergency CS rates than locals |
| 11.Merten S et al.,2007 | SWITZERLAND | *N=363;Peru and Dominican Republic* | Quantitative-CS | YES | CS use, birth weight, BFI, PTB, TIC | CS (37%) > locals (26%) and most migrant groups  Birth weight (3,276gr) ~ locals (3,278gr)  BFI (~97%) > locals (92%)  PTB in Peruvians (4%) < locals (9%)  PTB in Dominicans (11%) > locals (9%)  TIC in Peruvians( 5%) < locals (8%) |
| 12.Nedstrand E et al.,1995 | SWEDEN | *N=49;vc mainly Chile* | Quantitative-CS | NO | Age at menopause | Age at menopause (47) < locals (50) |
| 13.Ny P et al.,2007 | SWEDEN | *N=73;vc* | Quantitative-CS | NO | Use of ante-natal care services | Unlike all other migrant groups, HAs did not use antenatal care either less or later than recommended |
| 14.Pérez-Alcalá I et al.,2013a | SPAIN | *N=301;vc* | Quantitative-CS | NO | Age at menopause | Age at menopause (50.5) < locals (52) |
| 15.Pérez-Alcalá I et al.,2013b | SPAIN | *N=301;vc* | Quantitative-CS | NO | Reported hot flashes | HAs less likely to report hot flashes than locals |
| 16.Puig Sola C et al.,2008 | SPAIN | *N=205;vc* | Quantitative-CS | NO | LBW, PTB, Perinatal mortality, risk of infection | LBW (4%) < locals (10%); PTB (9%) < locals (18%)  Risk of infection (73%) > locals (62%)  Poor pre-natal care (9%) > locals (6%) |
| 17.Restrepo-Mesa SL et al.,2010 | SPAIN | *N=17,456;Colombia* | Quantitative-CS | NO | LBW | LBW (2.1%) < locals (3.4%) |
| 18.Río I et al.,2010a | SPAIN | *N=6,232;vc* | Quantitative-CS | NO | CS use, LBW | LBW (4%) < locals (6%); CS in HAs > locals |
| 19.Río I et al.,2010b | SPAIN | *N=931;vc* | Quantitative-CS | NO | BW, LBW, very LBW | Average BW (3.346gr) > locals (3.221gr)  LBW (5.7%) ~ locals (5.8%);  Very low BW (0.7%) > locals (0.6%) |
| 20.Río I et al.,2011 | SPAIN | *N=15,799;vc* | Quantitative-CS | NO | BFI | BFI (92%) > locals (80%) |
| 21.Robertson E et al.,2005 | SWEDEN | *N=2,143;vc* | Quantitative-CS | NO | NNB | Risk of NNB 50% higher than in Swedish |
| 22.Sampedro A et al.,2010 | SPAIN | *N=113;vc* | Quantitative-CS | NO | T. Gondii/T. Pallidium prevalence | T.Gondii in pregnancy (49%) > locals (14%)  T.Pallidum in pregnancy (3.5%) > locals (0.07%) |
| 23.Saurina C et al.,2012 | SPAIN | *N=163;n/a* | Quantitative-CS | NO | Use of FP | More likely to use FP methods than SSA and Asians |
| 24.Urquia ML et al.,2010 | VC | *N=67,788;vc* | Systematic review | NO | LWB, PTB | LBW (6.2%) > native born (4.3%) |
| 25.Vangen S et al.,2000 | NORWAY | *N=1,466;vc* | Quantitative-CS | NO | CS use, LBW | CS (24%) > locals (12%); LBW (3.6%) < locals (4.5%) |
| 26.Vikanes A et al.,2008 | NORWAY | *N=5,073;vc* | Quantitative-CS | NO | HG | Decrease risk of HG with increased length of residence |
| 27.Wolff H et al.,2005 | Switzerland | N=104;vc | Quantitative-CS | NO | Unintended pregnancies, cervical smear uptake | Four out of five pregnancies resulting in live births were unintended in irregular women  Underutilisation of cervical smear tests |

Acronyms used: CO (country of origin); vc (various countries); CS (cross-sectional); PTB (pre-term birth); LBW (low birth weight); SA (South American); CA (Central America & Caribbean); CS (caesarean section); *HAs (Hispano Americans);* EDA (epidural analgesia); GDM (gestational diabetes mellitus); FP (family planning); EE(East Europeans); BFI (breast feeding initiation); TIC (transfer to intensive care); NNB (non-normal birth); SSA (Sub-Saharan Africans); HG (hyperemesis gravidarum)
