# Supplementary material for: Hispano-Americans in Europe: what do we know about their health status and determinants? A scoping review
Source: BMC Public Health. 2015 May 7;15:472. doi: 10.1186/s12889-015-1799-x (PMC4430018; doi:10.1186/s12889-015-1799-x)
Supplement: Additional file 13: — Studies on health services. [file 12889_2015_1799_MOESM13_ESM.doc]

**Additional file 13. Studies on health services**

| **Study reference** | **Location** | **Participants**  ***N;CO*** | **Study design** | **Trans-**  **national** | **Outcome measure** | **Key findings** |
| --- | --- | --- | --- | --- | --- | --- |
| 1.Albares Tendero MP et al.,2008 | SPAIN | *N=641;vc* | Quantitative-CS | NO | Use of dermatology services | Use of dermatology services in HAs (8.5%) < locals (16.2%) > other migrant |
| 2.Bermudez EM,2004 | SPAIN | *N=17;Colombia* | Qualitative | NO | Users’ and providers’ perceptions of health services | Overall satisfaction with services. Reported quality of services compared to country of origin rated as excellent. Emergency services preferred for practical reasons |
| 3.Binfa L et al.,2010 | SWEDEN | *N=24;Chile* | Qualitative | NO | Perceptions about health providers | Power imbalances between health care providers and users persist  Providers’ communication skills should improve |
| 4.Brigidi S et al.,2008 | ITALY | *N=3,832;vc,*  *mainly Ecuador* | Mixed methods  (Quantitative + Qualitative) | NO | Use of emergency services, reasons for consultation | Emergency services tend to be used for non-urgent conditions when no entitlements to access primary care. Main reasons for consultations: abdominal pains, gynaecological and obstetrician problems, fever and alcoholism. Satisfaction with medical system *vs* country of origin |
| 5.Esteban y Peña MM,2001 | SPAIN | *N=405;n/a* | Quantitative-CS | NO | Reasons for consultation | Main reasons for consultation (excluding obstetric/gynaecological): respiratory infections, depression, headaches, back pain |
| 6.Fernández-Castillo A et al.,2009 | SPAIN | *N=14;n/a* | Qualitative | NO | Professionals’ perceptions | HAs presented *“less difficulties”* than other migrants due to linguistic and “cultural affinity”. Protocols to attend foreigners poorly known and applied |
| 7.Hjern A et al.,2000 | SWEDEN | *N=548;Chile* | Quantitative-CS | NO | Access to dental care | Chileans poor dental health > locals but three time less likely to access care |
| 8.López Nicolás A et al.,2009 | SPAIN | *N=81,423;n/a* | Quantitative-CS | NO | Use of health services | Use of emergency/hospital services in HA women aged 20-40 > locals  Use of emergency services in HA men < locals. When demographic factors are taken into account, use of emergency services in HAs < locals |
| 9.Muñoz de Bustillo R et al.,2010 | SPAIN | *N=~800;n/a* | Quantitative-CS | NO | Use of health services | No differences in pattern of use of health services in HAs *vs* locals except for higher use of emergency rooms by the former |
| 10.Ortell Ros E et al.,2011 | SPAIN | *N=16,272;n/a* | Quantitative-CS | NO | Reasons for consultation | Most reasons for consultation fall in the "Pregnancy and Childbirth" category (44%), followed by digestive problems (11%) and trauma (7%). Infectious diseases: 3.2% of all cases |
| 11.Recasens Oliva E et al.,2008 | SPAIN | *N=26;n/a* | Quantitative-CS | NO | Application of clinical protocols | Immunized HA children: 70%. HA children attended within first month after arrival: 80% |
| 12.Sanz B et al.,2011 | SPAIN | *N=1,702;n/a* | Quantitative-CS | NO | Use of health services | Use of emergency services in HA men > locals  Use of emergency services in HA women ~ locals  Use of specialist services in HAs < locals |
| 13.Saurina C et al.,2012 | SPAIN | *N=107;n/a* | Quantitative-CS | NO | Use of health services | Higher likelihood of first contact with health services in HA with cholesterol |
| 14.Taberner R et al.,2010 | SPAIN | *N=457;vc* | Quantitative-LN | NO | Use of dermatology services | HAs consultation for dermatology services (1.8%) < locals (2.7%)  Low incidence of tropical and imported diseases |
| 15.Terraza Nuñez R et al.,2010 | SPAIN | *N=18;Ecuador* | Qualitative | NO | Users’ perceptions regarding access to health services | Access to health services perceived as easy after obtaining a personal health-care card but insufficient information on process to obtaining it  Barriers to health service use: fear of “being recorded”, losing jobs and mistrust in health providers. Resort to formal care when illness worsens |

*Acronyms used: CO (country of origin); vc (various countries); CS (cross-sectional); HAs (Hispano Americans); HA (Hispano American); n/a (not available); LN (longitudinal)*
