# Supplementary material for: Hispano-Americans in Europe: what do we know about their health status and determinants? A scoping review
Source: BMC Public Health. 2015 May 7;15:472. doi: 10.1186/s12889-015-1799-x (PMC4430018; doi:10.1186/s12889-015-1799-x)
Supplement: Additional file 14: — Studies on social, economic and occupational factors. [file 12889_2015_1799_MOESM14_ESM.doc]

**Additional file 14. Studies on social, economic and occupational factors**

| Study reference | Location | *Participants*  ***N;CO*** | Study design | Trans-  national | Main factors addressed | Key findings |
| --- | --- | --- | --- | --- | --- | --- |
| 1.Agudelo-Suárez A et al.,2008 | SPAIN | *N=21;Colombia* | Qualitative | NO | Working conditions | Health problems perceived as closely linked to living and working conditions. Poor information on how to prevent work related hazards. Poor emotional health (“immigrant syndrome”) |
| 2.Benitez Robredo T et al.,2004 | SPAIN | *N=143;vc,mainly Ecuadorians* | Quantitative-CS | NO | Living and working conditions | Women mostly employed in domestic services under irregular precarious conditions. Relatively high educational levels |
| 3.Ceuterick M et al.,2011 | UK,BOLIVIA, PERU | *N=98;Bolivia,Peru* | Qualitative | YES | Use of traditional herbal remedies | Herbal remedies used for similar symptoms in Europe and at countries of origin (gastrointestinal/respiratory). Remedies for fatigue/headache more commonly used by Bolivians in London *vs* Cochabamba |
| 4.Colorado-Yohar S et al.,2012 | SPAIN | *N=672;n/a* | Quantitative-CS | NO | Exposition to violence,  perceived discrimination | Prevalence of violence (6.5%) > locals (2.7%), associated with perceived discrimination and low educational levels |
| 5.Dunlavy A et al.,2013 | SWEDEN | *N=407;n/a* | Quantitative-CS | NO | Working conditions | Exposure to adverse working conditions affect minimally the risk of poor health in employed HAs working an average of 38 hours/week |
| 6.Fuente A et al.,2012 | SPAIN | *N=407;vc* | Quantitative-CS | NO | Social integration, perceived discrimination, community support | Discrimination negatively related with social integration. Association disappears if informal community support considered |
| 7.Gonzalez-López JR et al.,2012 | SPAIN | *N=190;vc* | Quantitative-CS | NO | Self-medication and its determinants | Self-medication with anti-inflammatory drugs, analgesics and antibiotics > other migrant groups. Length of residence and use of traditional remedies positively related with self-medication |
| 8.Rodriguez Alvarez E et al.,2008 | SPAIN | *N=167;n/a* | Quantitative-CS | NO | Perceived health status and determinants | Men scored higher in perceived health status than women  HAs assessing their health as good/very good: 66% |
| 9.Sanchón-Macias MV et al.,2013 | SPAIN | *N=371;vc* | Quantitative-CS | NO | Subjective social status, perceived health | Those at the lowest levels of subjective social status assessed their perceived health as negative more often. Subjective social status predicts health better that socio-economic measures |
| 10.Salinero-Fort MA et al.,2012 | SPAIN | *N=691;vc* | Quantitative-CS | NO | Perceived health status and determinants | Self-reported health status < locals. Better self-reported heath associated to being man, < 34 years, upper-middle socioeconomic status, high social support and low stress.  Length of stay negatively related to self-reported health |
| 11.Sundquist J,1995a | SWEDEN, Chile, Uruguay | *N=398; Chile/Uruguay* | Quantitative-CS | YES | Perceived health status and determinants | Being a HA refugee is an independent factor for poor self-reported health. HAs had less social contacts, were more likely to live in rented flats, and had lower material standards than locals. Not feeling secure in day-to day life and poor leisure opportunities related to poor self-reported health. Often worked in occupations below their educational level. Being an immigrant is a risk factor for poor health |
| 12.Sundquist J,1995b | SWEDEN | *N=398;vc* | Quantitative-CS | NO | Perceived health status and determinants | Strong association between being a HA refugee and ill-health.  Ethnicity is an independent risk indicator for long term illness after controlling for social class, material standards, sex, age |
| 13.Villaroel N and Artazcoz L,2012 | SPAIN | *N=1,103;vc* | Quantitative-CS | NO | Perceived health status | People from Bolivia had the poorer heath outcomes (particularly Bolivian males). People form Argentina and Colombia had the best health outcomes. Length of residence, ethnicity, residency status, and length of migration are factors to consider |
| 14.Wright K,2011 | UK | *N= 49;Peru* | Qualitative | NO | Life satisfaction | Material and non-material variables including perceived exclusion, lack of social cohesion and feelings of low personal autonomy are important aspects of life satisfaction |

*Acronyms used: CO (country of origin); vc (various countries); CS (cross-sectional); n/a (not available); HAs (Hispano Americans); HA (Hispano American)*
