# Supplementary material for: Hispano-Americans in Europe: what do we know about their health status and determinants? A scoping review
Source: BMC Public Health. 2015 May 7;15:472. doi: 10.1186/s12889-015-1799-x (PMC4430018; doi:10.1186/s12889-015-1799-x)
Supplement: Additional file 15: — Studies on health behaviours. [file 12889_2015_1799_MOESM15_ESM.doc]

**Additional file 15. Studies on health behaviours**

| Study reference | Location | Participants  ***N;CO*** | Study design | Trans-  national | Health behaviour | Key findings |
| --- | --- | --- | --- | --- | --- | --- |
| 1.Codesal DM et al.,2010 | UK,SPAIN, USA, ECUADOR | n/a;Ecuador | Mixed methods  (Qualitative and Quantitative) | YES | DIET | The impact of migration on eating practices depends on distance from country of origin, legal status restricting more or less return visits at home, price and availability of local (or similar) ethnic foods, and social networks |
| 2.Gilbert PA et al.,2008 | EUROPE | Not applicable | Literature review | NO | DIET | Decreased consumption of fruits/soups and increased in processed foods |
| 3.González López JR et al.,2010 | SPAIN | N=33;vc | Quantitative-CS | NO | SUBSTANCE USE,PHYSICAL ACTIVITY | Participants consuming alcohol weekly: 38%; using illegal drugs last 6 months: 6%; smoking daily: 3%; self-medicating: 60%; physically inactive: 68% |
| 4.González López JR et al.,2012 | SPAIN | N=187;vc | Quantitative-CS | NO | SUBSTANCE USE | Participants consuming alcohol last month: 61%; smokers: 26%; consuming illegal substances in the last 6 months: 5.3%; mean age start smoking in HAs > locals |
| 5.Marsiglia FF et al.,2008 | SPAIN | N=181;vc | Quantitative-CS | NO | SUBSTANCE USE | HAs reported lower levels of use/intention to use illicit drugs vs Spanish |
| 6.Monras M et al.,2006 | SPAIN | N=22;n/a | Quantitative-CS | NO | ALCOHOL CONSUMPTION | Very low compliance to group therapy in HA patients with alcohol addiction (76% HAs abandoned therapy *vs* 54% locals) |
| 7.Posada E et al.,2011 | SPAIN | N=74;Bolivia | Qualitative | NO | DIET | Dietary changes influenced by practical and labour-related factors like availability of specific products and time to cook. Reduced consumption of soup, fruits and drinks |
| 8.Romo R et al.,2012 | SPAIN | N=262;vc | Quantitative-CS | NO | DIET | The higher the level of ethnic identity and feeling of belonging the greater the persistence of dietary habits from the country of origin |
| 9.Tordable Merino I et al.,2010 | SPAIN | N=58;vc | Quantitative-CS | NO | ALCOHOL CONSUMPTION, SUBSTANCE USE | Substances most commonly used by HAs: cocaine and alcohol |
| 10.Tortajada S et al.,2008 | SPAIN | N=757;vc | Quantitative-CS | NO | ALCOHOL CONSUMPTION, SUBSTANCE USE | Participants consuming alcohol habitually: 40%; cannabis: 3.4%. Licit drugs accepted but illegal drugs seen as dangerous |
| 11.Tortajada S et al.,2010 | SPAIN | N=610;vc | Quantitative-CS | NO | ALCOHOL AND CIGARETTE CONSUMPTION | Respondents reporting to drink more alcohol in Spain than in their country of origin: 38%. Current smokers: 30%. Alcohol consumption did not vary with length of stay (< 2 years *vs* > 2 years) and perceived discrimination |

Acronyms used: *CO (country of origin);* n/a (not available), *vc (various countries); CS (cross-sectional); HAs (Hispano Americans); HA (Hispano American)*
