# Supplementary material for: Hispano-Americans in Europe: what do we know about their health status and determinants? A scoping review
Source: BMC Public Health. 2015 May 7;15:472. doi: 10.1186/s12889-015-1799-x (PMC4430018; doi:10.1186/s12889-015-1799-x)
Supplement: Additional file 17: — Studies reviewed by topic addressed. [file 12889_2015_1799_MOESM17_ESM.doc]

**Additional file 17. Studies reviewed by topic addressed**

Source: Authors
